# ERK mediates interferon gamma-induced melanoma cell death

**DOI:** 10.1186/s12943-023-01868-x

**Published:** 2023-10-06

**Authors:** Ameya Champhekar, Rachel Heymans, Justin Saco, Guillem Turon Font, Cynthia Gonzalez, Anne Gao, John Pham, June Lee, Ryan Maryoung, Egmidio Medina, Katie M. Campbell, Daniel Karin, David Austin, Robert Damioseaux, Antoni Ribas

**Affiliations:** 1 Division of Hematology-Oncology, Department of Medicine, University of California, Los Angeles, Los Angeles, CA 90095 USA; 2 Department of Molecular and Medical Pharmacology, University of California, Los Angeles, CA 90095 USA; 3 California NanoSystems Institute, University of California, Los Angeles, CA 90095 USA; 4 Jonsson Comprehensive Cancer Center, Los Angeles, CA 90095 USA; 5 Department of Bioengineering, Samueli School of Engineering, University of California, Los Angeles, CA 90095 USA; 6 Division of Surgical Oncology, Department of Surgery, University of California, Los Angeles, Los Angeles, CA 90095 USA; 7 Parker Institute for Cancer Immunotherapy, San Francisco, CA 94129 USA

**Keywords:** IFNγ, ERK signaling, Tumor growth inhibition, Stress response, Melanoma

## Abstract

**Background:**

Interferon-gamma (IFNγ) exerts potent growth inhibitory effects on a wide range of cancer cells through unknown signaling pathways. We pursued complementary screening approaches to characterize the growth inhibition pathway.

**Methods:**

We performed chemical genomics and whole genome targeting CRISPR/Cas9 screens using patient-derived melanoma lines to uncover essential nodes in the IFNγ-mediated growth inhibition pathway. We used transcriptomic profiling to identify cell death pathways activated upon IFNγ exposure. Live imaging experiments coupled with apoptosis assays confirmed the involvement of these pathways in IFNγ-mediated cell death.

**Results:**

We show that IFNγ signaling activated ERK. Blocking ERK activation rescued IFNγ-mediated apoptosis in 17 of 23 (~ 74%) cell lines representing BRAF, NRAS, NF1 mutant, and triple wild type subtypes of cutaneous melanoma. ERK signaling induced a stress response, ultimately leading to apoptosis through the activity of DR5 and NOXA proteins.

**Conclusions:**

Our results provide a new understanding of the IFNγ growth inhibition pathway, which will be crucial in defining mechanisms of immunotherapy response and resistance.

**Supplementary Information:**

The online version contains supplementary material available at 10.1186/s12943-023-01868-x.

## Background

The cytokine interferon-gamma (IFNγ) is a major effector of antitumor immunity. It is produced predominantly by activated T and NK cells and exerts multiple effects on tumor cells. Signaling through the IFNγ receptor triggers a tumor cell gene expression program that amplifies the antitumor immune response [1]. This includes increased expression of the chemokines CXCL9/10/11, which lead to the recruitment of more immune cells to the tumor site. Tumor cells also upregulate the expression and activity of antigen-processing and presentation machinery genes related to both MHC I and II pathways, effectively increasing tumor visibility to the immune system [2, 3].

Besides these effects, IFNγ directly inhibits tumor cell growth through antiproliferative and proapoptotic activity. The initial description of interferons (and their name) was related to interference with the growth of virally-infected cells [4]. However, the specific pathway that bridges IFNγ signaling with cellular growth inhibition is not fully characterized. The IFNγ receptor is comprised of two subunits, IFNGR1 and IFNGR2, which are associated with JAK1 and JAK2 kinases, respectively. The binding of IFNγ dimers to the receptor complex activates JAK1 and JAK2 kinases, which phosphorylate STAT1. Phosphorylated STAT1 homodimers translocate to the nucleus and induce the expression of primary response genes, including the transcription factor IRF1. IRF1, in turn, regulates the expression of several secondary response genes that together make up the characteristic IFNγ gene expression signature [5]. Early studies indicated an indispensable role for the transcriptional activity of both STAT1 and IRF1 in inducing tumor growth inhibition. Thus, IFNγ was shown to upregulate the expression of the cell cycle inhibitor p21 and cell death effectors, including Caspase 1, 3, and 8 [6, 7]. Other studies reported upregulation of FAS and FAS ligand [8] and TRAIL expression [9], which resulted in cell death. In contrast, a later study with melanoma cells found that IFNγ upregulated p21 and p27, which were not responsible for cell cycle inhibition [10]. Instead, their results implicated the downregulation of Cyclin A and E, which regulate G1-S cell cycle transition, in the growth arrest observed after IFNγ treatment. Finally, one study also demonstrated that IFNγ signaling led to RIP1-mediated necroptosis [11].

Despite this progress, the relative contribution of these pathways to growth inhibition remains unknown. Additionally, the lack of mutational profiles of the lines used in these early studies makes it hard to determine whether a particular mode of growth inhibition generally applies to all subtypes. Apart from the canonical JAK-STAT pathway, IFNγ is known to activate other signaling proteins, including the Src-family kinase Fyn, adaptors like c-Cbl and Vav [12], Pyk2, and MAPKs ERK1/2 [13, 14]. However, the impact of these pathways on the growth-inhibitory effects of IFNγ is not known.

Here we used complementary screening approaches to delineate the signaling pathway leading to the growth inhibition of melanoma cells. Our results identified ERK as a major downstream target of IFNγ signaling that is crucial for this process. We show that ERK is activated following IFNγ treatment, and activated ERK leads to the induction of cell death through a pathway involving the upregulation of a stress response program. Our results identify novel aspects of the IFNγ growth inhibition pathway that will be crucial to understanding resistance mechanisms.

## Methods

### Cell culture

All patient-derived melanoma lines were cultured in RPMI-1640, supplemented with 10% FBS, 10mM HEPES, and penicillin, streptomycin, and amphotericin B. Cell lines were authenticated periodically using the GenePrint10 system (Promega). Cultures were tested for mycoplasma contamination every 3–4 months using the MycoAlert Mycoplasma Detection Kit (Lonza).

### CRISPR screen

M238 cells were transduced with the LentiCas9-Blast [15] virus, and clonal Cas9-expressing lines were established after a week of blaticidin selection. A single clonal line with an IFNγ sensitivity profile similar to the parental M238 line was used to set up the screen. Cas9-expressing M238 cells were transduced with human GeCKO v2 [15] or Brunello [16] sgRNA libraries (MOI of ≤ 0.3), selected for a week with Puromycin, and divided into 2 treatment groups, each resulting in ≥ 500X sgRNA library representation. One group was treated with IFNγ (Peprotech, 800 U/ml), while the other was left untreated (control). Culture media was replaced with fresh IFNγ-containing media every 3–4 days. Cells were harvested between days 10–14 of selection, genomic DNA was extracted and library preparation was performed as described previously [17]. Libraries were sequenced on an Illumina NextSeq500 instrument at the UCLA Technology Center for Genomics and Bioinformatics (TCGB). MAGeCK software was used to obtain counts and calculate differential sgRNA enrichment.

### Western blotting

Unmodified or shRNA-expressing melanoma lines were treated with or without IFNγ (100 U/ml) for up to 72 h. For serum starvation experiments, cells were cultured in low-serum media containing 0.5% FBS for 48 h before starting IFNγ stimulation. Western blotting was performed as described previously [18]. Band intensities were quantified using the Fiji software. Blots were probed with DR5, total and phospho STAT1, ERK1/2, GAPDH (Cell Signaling) and NOXA (Novus Biologicals) antibodies. A list of antibodies and reagents is included in Supplementary Table 1.

### RNA-Seq analysis

M230 and M238 cells were treated with DMSO (control), human IFNγ (100 U/ml, Peprotech), and/or ulixertinib (Selleckchem, 6 µM) for 24 h. Total RNA was extracted, and library preparation was performed at the UCLA TCGB core using KAPA Stranded RNA-Seq with RiboErase Kit, and single-end sequencing (1 × 50 bp) was performed on Illumina HiSeq3000 instrument. Reads were mapped to the GRCh38 reference genome using HISAT2, and HTseq-counts, and Stringtie were used to obtain read counts, and FPKM values, respectively. Read count data were used to perform differential expression analysis with the DESeq2 package. A threshold of adjusted *P* ≤ 0.05 and fold change ≥ 2 were applied to define differentially expressed genes for each pairwise comparison. Enrichment analyses for Reactome pathways and various MSigDB genesets were performed using the ClusterProfiler package.

### Incucyte experiments

Selected lines were plated overnight at 2000–5000 cells/well in clear 96-well plates (TPP) in 100 µl media, and the indicated drugs, IFNγ (Peprotech) and the Cytotox Red dye (Sartorius), were added on the next day in triplicate wells per condition. Ulixertinib treatment concentrations were determined for each melanoma line by testing a range of doses between 25 and 2000 nM and selecting the highest dose that did not affect cell growth or induce cell death. Plates were immediately transferred to the IncuCyte instrument, and images were acquired every 2 h for 7 days. Area under the curve (AUC) was calculated, and heatmaps were generated using the R packages DescTools and pheatmap, respectively.

For Caspase 3/7 activity experiments, cell plating and treatments were as above. ISRIB (Selleckchem) was added at 1 µM while Z-IETD-FMK (Selleckchem) was used at 50 µM concentrations. On day 5 of treatment, Nuclear ID Red (Enzo life science) and the CellEvent Caspase 3/7 Green reagent (ThermoFisher Scientific) were added to the wells at 1:1000 dilution each, and plates were imaged in a IncuCyte Zoom instrument after 2 h. Normalized caspase 3/7 activity/mm^2^ was calculated by dividing the number of events with overlapping caspase 3/7 activity and nuclear staining/mm^2^ with the total number of nuclear-stained objects/mm^2^.

**Fig. 1:**
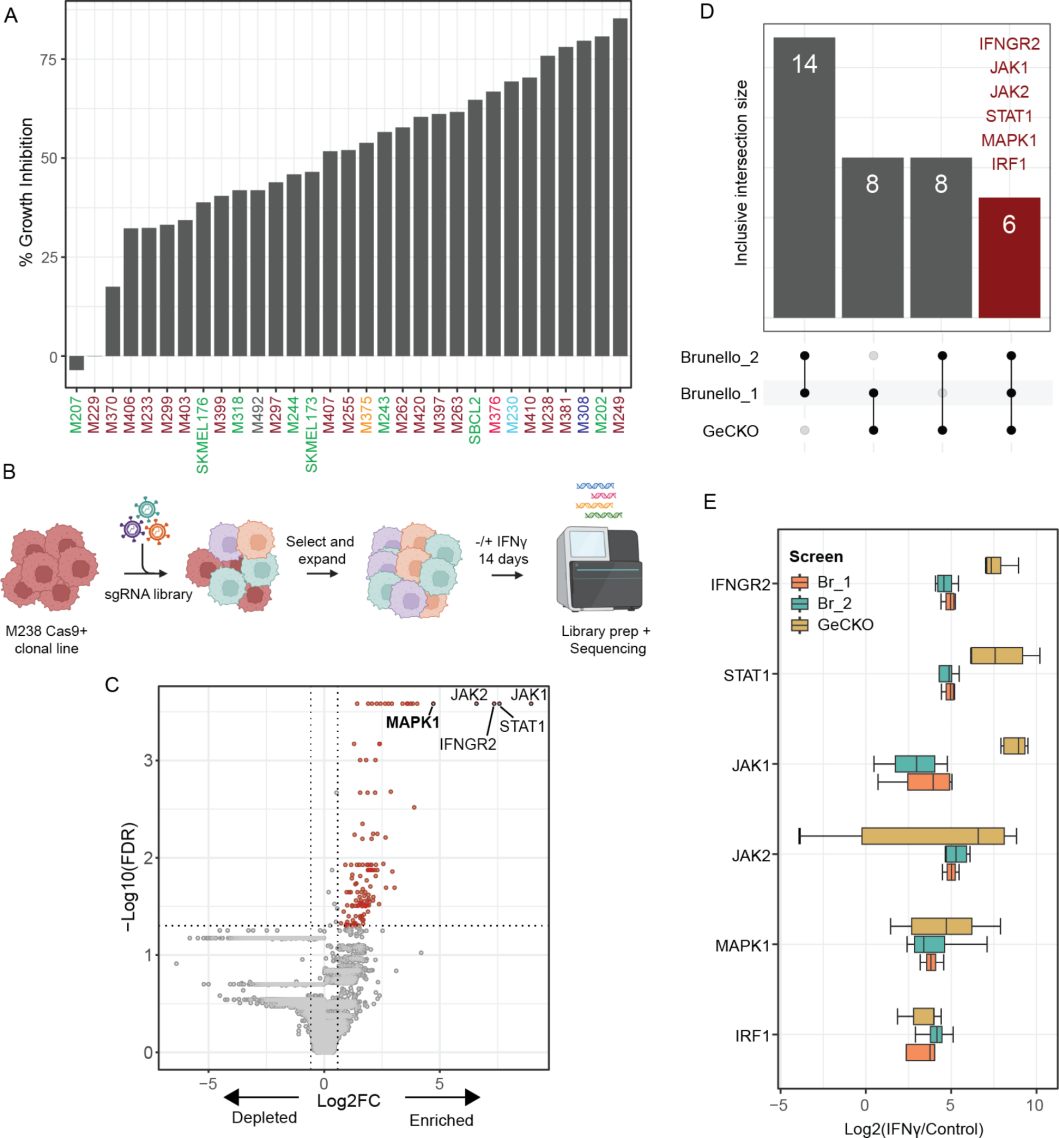
CRISPR screen uncovers an essential role for ERK2 in IFNγ-mediated growth inhibition. **(A)** Melanoma lines were treated with or without IFNγ in triplicate, and cell growth was monitored for 7 days in an IncuCyte live imaging experiment. Line names are color-coded to indicate melanoma subtypes: Maroon, BRAF mutant; Green, NRAS mutant; Cyan, NF1 mutant; Orange, triple wild type; Red, BRAF and NRAS double mutant; Dark blue, BRAF and NF1 double mutant; Black, not determined. Refer to Supplementary Table 2 for cell line details. Growth curves for selected lines are presented in Fig. S1. **(B)** Schematic depicting the experimental setup for all three CRISPR screens. **(C)** Representative volcano plot with results from the GeCKO library. Fold enrichment or depletion in the IFNγ-treated sample calculated over all sgRNAs targeting each gene is shown on the x-axis, while the y-axis shows the statistical significance for each gene. Top 5 enriched genes are labeled on the plot. **(D)** Lists of the top 30 genes enriched in the IFNγ-treated sample for all three screens were compared to identify top hits common to all three screens. **(E)** Plot showing fold enrichment values for all the individual sgRNAs targeting ERK2 (MAPK1) and genes involved in the core IFNγ signaling pathway from each screen. ERK2 is enriched to a similar level as the IFNγ signaling genes across all three screens. See also Fig. S2

For Fig. 1A, Control and IFNγ-treated triplicate wells were imaged for 7 days. Average percent confluency data for control and IFNγ-treated cells at the first time point when the control cells reached max confluency was used to calculate percent growth inhibition using the formula: % Growth inhibition = 100 – ((avg. confluency IFNγ / avg. confluency Control) * 100).

### Drug screen

Drug screen was performed with M238 cells using a library of 3,265 compounds. Two identical sets of 384-well plates (Greiner Bio-One) were created by adding (1) 20 µl media/well, (2) 250 nl compounds in DMSO from each stock plate using a Biomek FX with V&P custom pin tool into columns 3–22 of two plates, and (3) 750 cells/well. Finally, 800 U/ml IFNγ solution was added to one set of plates, while the other received the same volume of media creating the Drug-only and Drug + IFNγ treatments. Each plate also had media with DMSO only (column 2, positive control) and 10,000 U/ml of IFNγ + DMSO (column 23, negative control) treated wells. Viability was measured after 96 h using the CellTiter-Glo assay (Promega). Hits from the pilot screen were tested in triplicate wells per condition in a confirmatory screen using the same procedure.

Raw data were uploaded to the Collaborative Drug Discovery vault (www.collaborativedrug.com) for analysis. Only plates with Z’ values of ≥ 0.5 were considered for analysis. Drugs with z > = 1.5 for the Drug + IFNγ condition but within the − 1.8 < z-score < 1.8 range for the Drug-only condition were selected as hits.

### Synergy experiments

Indicated concentrations of IFNγ, PMA, and M238 cells (750 cells/well) were tested in 4 wells/condition. Cell viability was determined on day 4 using the CellTiter-Glo assay (Promega). Percent viability was calculated compared to DMSO-treated control wells, and synergy was determined using SynergyFinder software.

### shRNA experiments

Two shRNAs, each targeting the human DR5 and NOXA genes, were cloned into the pLKO.5-puro vector and used to create stable shRNA expressing lines using M238 cells. An shRNA against GFP was used as a control. Each line was treated with IFNγ (100 U/ml) or left untreated (control) for 24 h. The extent of knockdown was determined by Western blotting.

### Statistical analysis

Caspase 3/7 activity data were analyzed using a two-tailed unpaired Student’s t-test to compare apoptosis levels between treatment groups. Specific comparisons are indicated in figure legends. A *P* < 0.05 was considered statistically significant, and statistically significant differences are denoted with asterisks. Differential gene expression was performed using the R package DeSeq2, which calculates differential expression based on a negative binomial generalized linear model fitting, and significance is determined by a Wald test. Adjusted P values were calculated using the Benjamini-Hochberg method.

## Results

### CRISPR and drug screens identify an essential role for ERK in IFNγ-mediated growth inhibition

We used a panel of 31 patient-derived melanoma lines to determine the extent of growth inhibition upon continued exposure to IFNγ (Fig. 1A and S1, and Supplementary Table 2). Our results indicated a heterogenous response ranging from complete resistance to a maximum of > 80% inhibition of cell growth, with an overall median of 52% growth inhibition. The two resistant lines (M207 and M229) did not harbor inactivating mutations in any of the core IFNγ-signaling genes. These data imply the presence of additional mechanisms through which cancer cells may resist growth inhibition by IFNγ and highlight the necessity to develop a better understanding of this pathway.

To define critical nodes in the IFNγ growth inhibition pathway, we set up a CRISPR screen using the GeCKO and Brunello whole-genome targeting sgRNA libraries. We used the *BRAF* V600E mutant M238 melanoma cell line from our test panel since it is highly sensitive to growth inhibition. We first established stable Cas9 nuclease expressing clonal lines and selected a clone that showed IFNγ sensitivity similar to the parental M238 line (Fig. S2). We set up three screens, one with the GeCKO and two with the Brunello library, using the same protocol (Fig. 1B).

We observed an enrichment of sgRNAs targeting core genes in the IFNγ sensing and signaling pathways (Fig. 1C-D). The IFNγ receptor 2 (*IFNGR2*), *JAK1*, and *JAK2* kinases, and *STAT1* were at the top of the list in all three screens. Additionally, the transcription factor *IRF1*, responsible for inducing several important IFNγ target genes, including PD-L1, was also among the top hits. Outside of the IFNγ signaling pathway, the only other hit enriched to the same magnitude was ERK2 (*MAPK1*) gene (Fig. 1E). We did not find *ERK1* sgRNAs among the top hits for any screen. ERK2 is expressed at higher levels in M238 cells than ERK1 (Fig. 3B and D). It is possible that the deletion of *ERK1* does not lead to a sufficient decrease in the total cellular ERK levels to affect the outcome of these screening experiments.

**Fig. 2:**
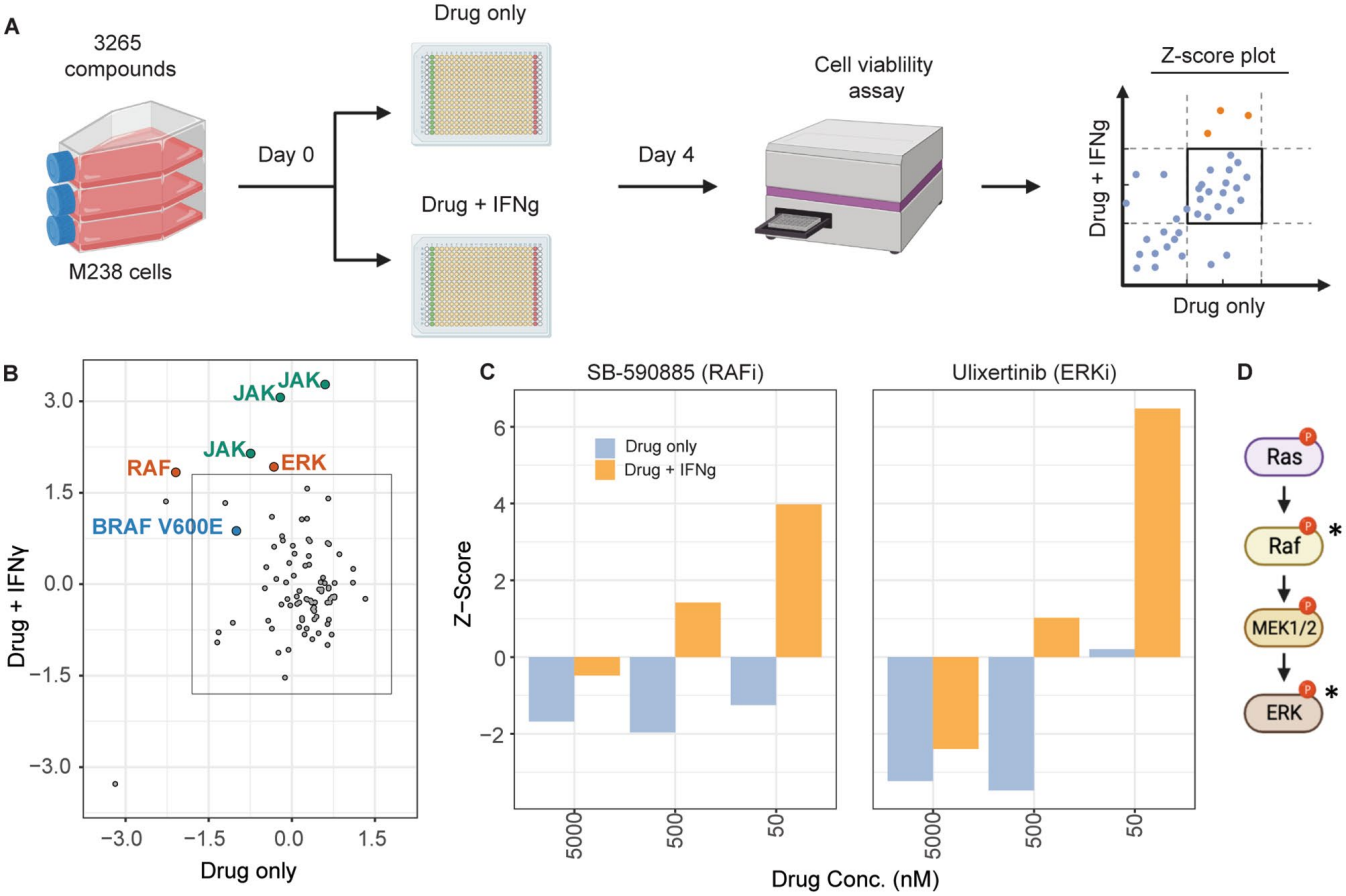
Drug screening identifies RAF and ERK as the mediators of IFNγ-induced growth inhibition. **(A)** Workflow for the drug screen. The orange dots on the Z-score plot indicate expected hits that rescue IFNγ-mediated growth inhibition but do not affect cell growth in the absence of IFNγ. **(B)** Z-score plot showing the hits from the confirmatory screen. Compounds that fall in the box that denotes a Z-score cutoff of 1.8 do not affect growth inhibition. Hits and targets of interest are labeled in colored text. Three separate JAK inhibitors were among the hits, as shown in green. Hits targeting the MAPK pathway proteins RAF (SB-590885) and ERK (BVD-523, ulixertinib) are shown in orange. BRAF V600E inhibitor Vemurafenib, labeled in blue, did not affect IFNγ growth inhibition. **(C)** Data from the pilot screen showing that inhibitors of RAF (left) and ERK (right) rescue growth inhibition in a dose-dependent manner. At lower doses, around 50 nM, both compounds can block IFNγ-mediated growth inhibition (orange bars). However, at higher doses, both compounds cause cell death in the presence (orange bars) or absence of IFNγ (blue bars). **(D)** Schematic showing the MAPK signaling cascade with the targets of drug screen hits marked with asterisks. See also Fig. S3

In parallel, we set up a screen with a targeted library of 3,265 compounds to identify drugs that block IFNγ-mediated growth inhibition (Fig. 2A). Our screen identified three different JAK kinase inhibitors as the top hits, thus validating the assay design (Fig. 2B). The only other hits in this screen were the compound SB590885, which inhibits the activity of BRAF, BRAF V600E, and CRAF kinases, and an ATP-competitive ERK inhibitor ulixertinib (BVD-523) [19]. A closer examination of the screening data revealed that both hit compounds showed a dose-dependent rescue of growth inhibition (Fig. 2C). Both drugs induced cell death at higher concentrations (> 500 nM), irrespective of the presence of IFNγ (blue bars). This was expected since the *BRAF* V600E mutant M238 line depends on constitutively active MAPK signaling for survival. At the lowest concentration (50 nM) that was well tolerated by the cells, both MAPK pathway inhibitors could rescue growth inhibition (orange bars). Taken together, the results from both screens indicate that ERK activity is essential for IFNγ-mediated growth inhibition. The ability of drugs to target multiple isoforms further revealed the involvement of the RAF-MEK-ERK cascade (Fig. 2D).

**Fig. 3:**
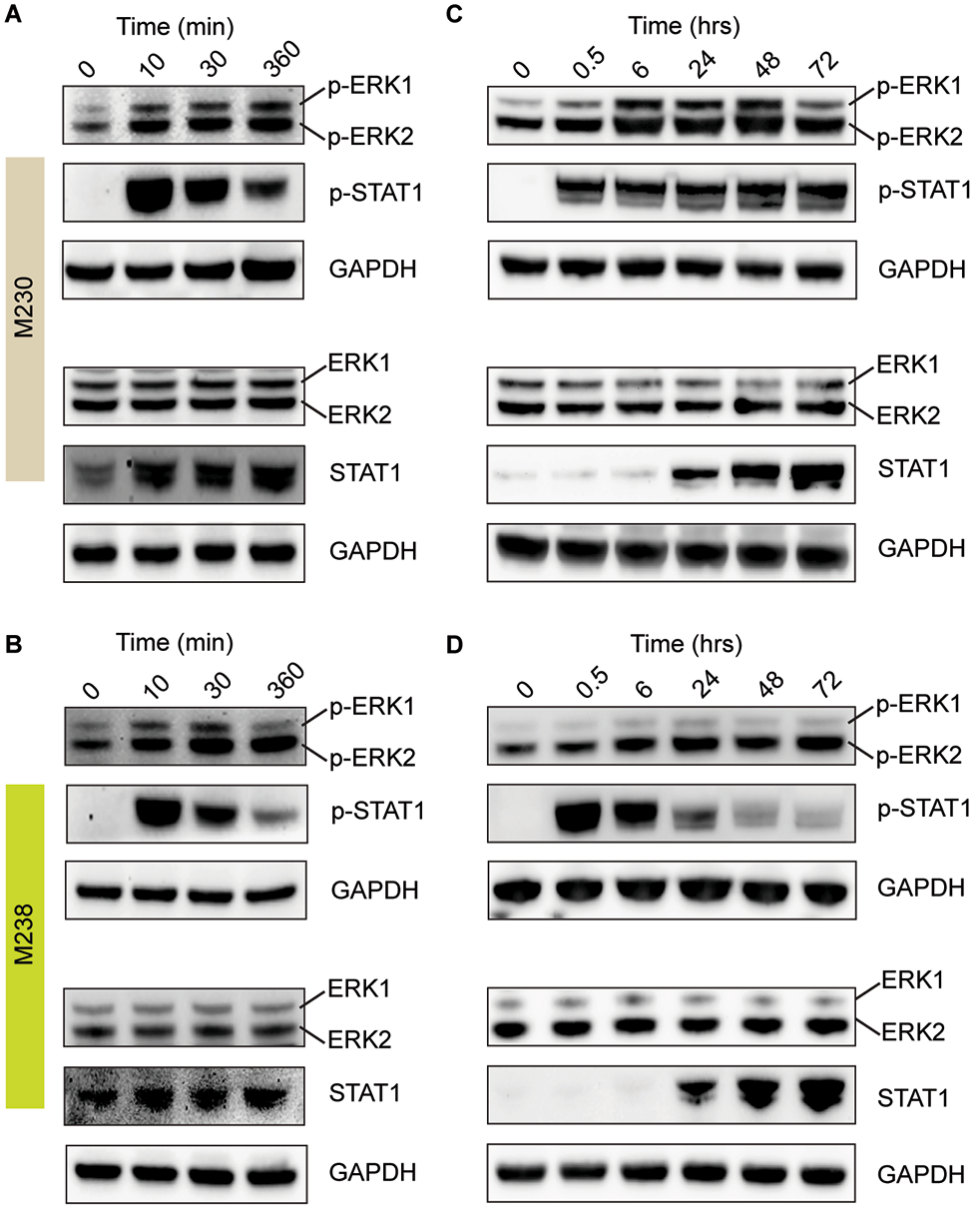
ERK is activated following IFNγ treatment. **(A)** M230 and **(B)** M238 cells were serum starved for 48 h, followed by IFNγ treatment (100 U/ml) for up to 6 h. The resulting samples were probed with antibodies indicated to the right. Results are representative of 3 independent experiments per line. **(C)** M230 and **(D)** M238 cells were treated with IFNγ (100 U/ml) for the indicated time and probed with various antibodies as labeled to the right. Results are representative of 2 and 3 independent experiments for the M230 and M238 lines, respectively. See also Figs S4-S7 for images of full blots for each experiment shown in this figure

### IFNγ signaling leads to ERK activation in melanoma cells

Since both RAF and ERK inhibitors could rescue growth inhibition, we investigated whether IFNγ exposure leads to ERK phosphorylation, which is required for its activation. We used the IFNγ-sensitive M238 (*BRAF* V600E mutant) and M230 (*NF1* mutant) lines for these experiments. M230 and M238 cells were serum-starved for 48 h to reduce basal ERK phosphorylation levels, followed by culture with IFNγ for 0–6 h (Fig. 3A and B, respectively). We subsequently increased IFNγ exposure to 72 h, this time without serum starvation, to test whether IFNγ induced sustained ERK phosphorylation (Fig. 3C-D). Results from both experiments showed a robust increase in p-STAT1 levels at the earliest time point, indicating the activation of JAK-STAT signaling as expected. An increase in p-ERK levels was observed at the earliest time point of 10 min, reaching peak levels between 24 and 72 h, indicating a sustained increase in ERK activity. Total ERK levels remained constant over the entire time course, indicating that IFNγ signaling increased ERK activity without affecting total protein expression. Our data thus indicate that IFNγ signaling results in ERK activation, and this event could be important in the growth inhibition pathway induced by IFNγ.

### ERK activity is essential for the induction of cell death after IFNγ exposure

**Fig. 4:**
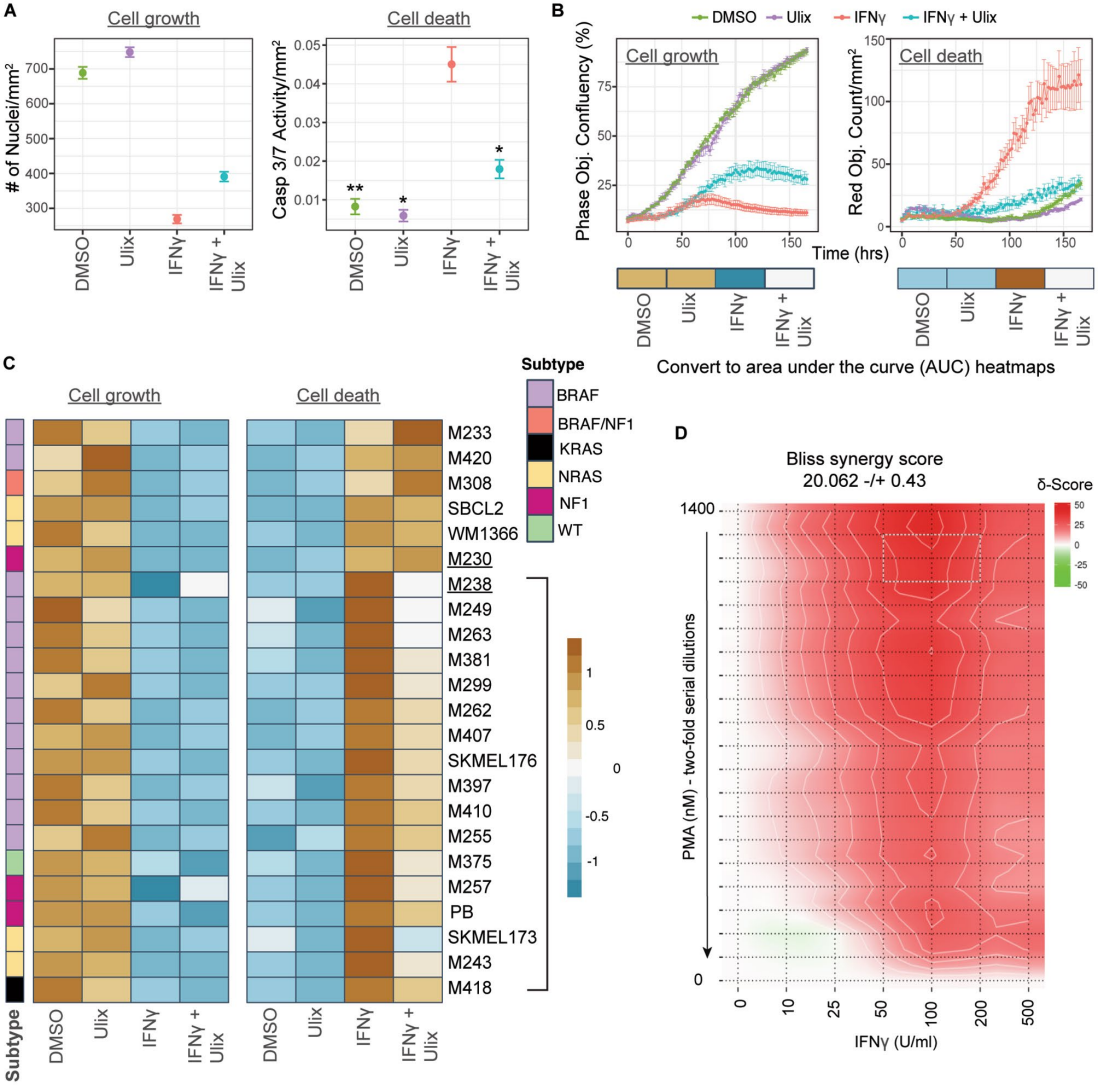
ERK activity is essential for cell death after IFNγ treatment. **(A)** M238 cells were treated in triplicate wells as labeled on the x-axis. Cells were stained on day 5 and imaged to enumerate total DNA-containing objects (left panel) and those with caspase 3/7 activity (right panel). Caspase activity counts were normalized by the total DNA-containing object counts, and means were plotted in the right panel. Error bars indicate SEM. Results are representative of 3 independent experiments. Statistically significant differences in cell death compared to the IFNγ-treated sample are indicated by an asterisk: * *P* < 0.05, ** *P* < 0.01. **(B)** M238 cells were plated with the indicated treatments (top) in triplicate wells per condition, along with the Cytotox Red dye, which stains dead or dying cells with compromised membrane integrity. Plots show changes in confluence (left panel) and dead cell count (right panel) over time. The area under the curve (AUC) was calculated for each curve and converted into a heatmap, as shown below in the respective plot. **(C)** Live imaging experiments, similar to panel B, were set up using 23 melanoma lines. AUC values were calculated as in B, and the resulting heatmaps are shown for cell growth (left) and cell death (right) curves for each line. The annotation to the left shows the molecular subtype of each tested melanoma line. The vertical bracket indicates 17 melanoma lines in which IFNγ-mediated cell death is rescued by ulixertinib treatment. Results are representative of 2 independent experiments. Heatmap labels for M230 and M238 lines, compared in an RNA-seq experiment in Fig. 5, are indicated with an underline. **(D)** M238 cells were treated with different IFNγ and PMA doses as indicated on the x and y axis, respectively, with four replicates per condition. The red color indicates areas of synergy between the two treatments with respect to growth inhibition. Area of maximum synergy is indicated with a box. Data is representative of 3 independent experiments. See also Fig. S8

We then determined whether ERK inhibition could block apoptosis by measuring Caspase 3/7 activity in IFNγ-treated cells. We used the ERK inhibitor ulixertinib at a concentration of 50 nM, which efficiently blocked growth inhibition in the drug screen but did not affect melanoma cell growth in the absence of IFNγ (Fig. 4A-B, left panels). Our results showed that ulixertinib almost completely rescued cell death (Fig. 4A, right panel; DMSO versus IFNγ and IFNγ plus ulixertinib). Inhibition of ERK activity only rescued cell death in these samples as cell counts remained low in samples cultured in both IFNγ and IFNγ plus ulixertinib (Fig. 4A-B, left panels, IFNγ versus IFNγ plus ulixertinib). These data indicate that ERK activity is essential for apoptosis induction downstream of IFNγ signaling. The magnitude of rescue validates our screening results and explains the similar level of enrichment of sgRNAs targeting ERK to those targeting proximal IFNγ signaling genes in the CRISPR screen.

Next, we tested whether the ERK-mediated cell death pathway was active in a larger panel of 23 melanoma cell lines (Fig. 4B-C, and Supplementary Table 2). This panel included cell lines representing all four molecular subtypes of cutaneous melanoma with driver mutations in the *BRAF*, *NRAS*, or *NF1* genes and a fourth triple wild-type subtype, in which none of these genes are mutated [20]. For each line, we first determined the highest ulixertinib concentration that did not affect cell growth compared to DMSO over 7 days of culture. Live imaging experiments were then set up in which cells were continuously monitored for 7 days in the presence of IFNγ with or without the chosen ulixertinib concentration. Results for the M238 line show that while IFNγ induced cell death as expected, ulixertinib completely blocked the induction of cell death (Fig. 4B, right panel). Cell growth, as measured by confluency, showed minimal rescue in line with previous results (Fig. 4B, left panel). We calculated the area under the curve (AUC) for each of the cell death and growth curves to summarize the data for all 23 cell lines and converted AUC values into a heatmap (Fig. 4C and Fig. S8A). Our results show that IFNγ-mediated cell death was rescued in 17 of 23 cell lines (~ 74%, rescued lines are indicated by a bracket). Notably, the rescued lines belong to all four molecular subtypes of cutaneous melanoma (Supplementary Table 2). These results indicate that the IFNγ-ERK cell death pathway is active in a majority of melanomas, irrespective of their mutational subtype. The diversity of our melanoma cell line panel also led to the discovery of some IFNγ-sensitive lines that were not rescued by ulixertinib exposure (e.g., M230, Fig. S9).

Finally, we also tested whether activation of ERK, using the known upstream ERK activator phorbol 12-myristate 13-acetate (PMA) [21], could enhance IFNγ-mediated growth inhibition. Our data show that PMA and IFNγ synergize over a wide range of dose combinations to significantly increase growth inhibition compared to either treatment alone (Fig. 4D and Fig. S8B). Taken together, our data establish the importance of ERK activation in the induction of cell death downstream of IFNγ signaling.

### ERK co-regulates the expression of several IFNγ response genes and induces an integrated stress response following IFNγ treatment

Next, we sought to understand which pathways downstream of ERK activation were involved in inducing cell death. We cultured M230 and M238 cells with IFNγ in the presence or absence of ulixertinib for 24 h and performed RNAseq analysis for four samples per line: DMSO, DMSO plus IFNγ, ulixertinib, and IFNγ plus ulixertinib (Fig. S10). These two lines were chosen for comparison because although IFNγ exposure activated ERK in both lines (Fig. 3), cell death was inhibited by ulixertinib only in M238 cells (Fig. S9).

**Fig. 5:**
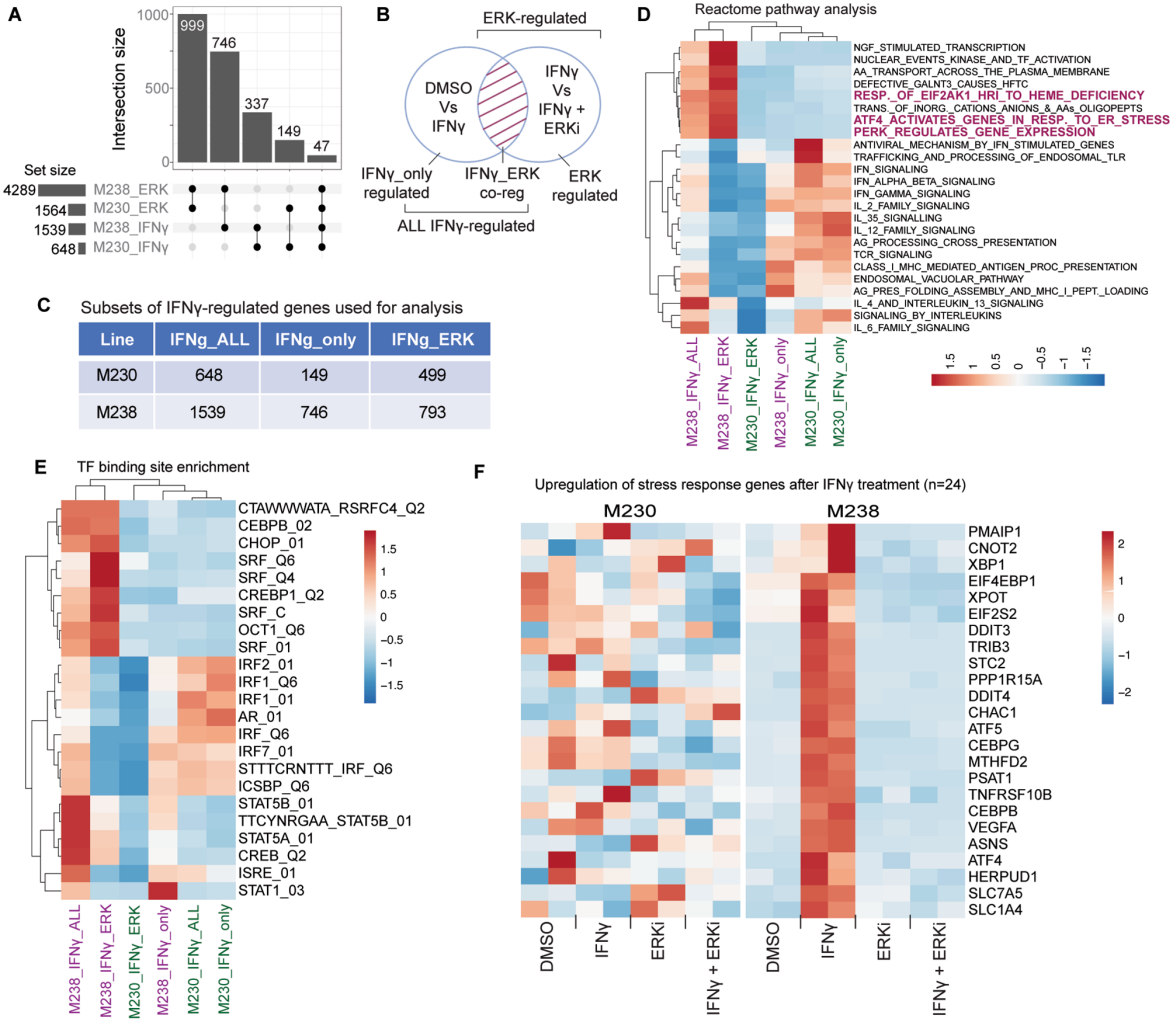
Comparison of IFNγ and ERK-induced gene expression for M230 and M238 melanoma lines. **(A)** Plot showing the overlap of different ERK-regulated (M230_ERK and M238_ERK) and IFNγ-regulated (M230_IFNγ and M238_IFNγ) gene sets defined by differential expression analysis. **(B)** Schematic showing how the different gene lists were derived for calculating overlaps and enriched gene sets. **(C)** Table showing the number of differentially expressed genes in each subset for the M230 and M238 melanoma lines. These gene sets were used to determine the pathway and transcription factor binding site enriched in each subset. Heatmaps were generated using the negative log10 of adjusted P-values for a union set of the top 10 enriched **(D)** reactome pathways and **(E)** transcription factor (TF) binding sites for each subset. All M230 gene sets are labeled in green and M238 in purple color text. **(F)** Heatmaps showing the FPKM expression values for 24 stress response genes for each sample in the M230 (left panel) and M238 (right panel) melanoma lines. The two columns per sample represent data from two independent experiments. See also Figs S10-S12 and Supplementary Table 3

Analysis of the RNA-seq data from two experiments revealed several differences between the two cell lines (Fig. 5A-B; Supplementary Table 3). We considered differentially expressed genes from two comparisons for each line, DMSO versus IFNγ, for genes regulated by IFNγ (Fig. 5A. M230_IFNγ and M238_IFNγ), and IFNγ versus IFNγ plus ulixertinib for all genes regulated by ERK in the presence or absence of IFNγ (Fig. 5A; M230_ERK and M238_ERK). Culture in ulixertinib identified ~ 3-fold more differentially expressed genes in the M238 line (4,289) than in the M230 line (1564). While ~ 1,000 genes were common to both cell lines, ERK regulated the expression of an additional extensive set of genes in the M238 cell line. Similarly, IFNγ regulated the expression of ~ 2.4 fold more genes in the M238 line (1,539) than in the M230 line (648). Of these, only 337 were common to both lines, again indicating the presence of a large set of genes regulated in a cell-specific manner.

For each line, we next defined a set of genes co-regulated by IFNγ and ERK as the overlap in the differentially expressed gene lists resulting from two comparisons, DMSO versus IFNγ, and IFNγ versus IFNγ plus ulixertinib (Fig. 5B; shaded area). This procedure subsets the IFNγ-regulated genes from the first comparison with the condition that they are also regulated by ERK, identifying genes regulated by both signaling pathways. Again, IFNγ and ERK regulated 746 genes in M238 cells, compared to only 149 in M230 cells (Fig. 5A). Of these, only 47 were common to both lines, indicating that ERK regulated many more genes in the M238 line downstream of IFNγ signaling. These results show that M238 cells may harbor a more permissive transcriptional state for the IFNγ and ERK pathways to regulate gene expression than the M230 line.

Next, we divided each set of IFNγ-regulated genes (Fig. 5B, ALL IFNγ-regulated, Fig. 5C, IFNγ_ALL) into two sub-sets based on their inferred regulation (i) IFNγ and ERK co-regulated genes (IFNγ_ERK) and (ii) those regulated by IFNγ without any contribution from ERK (IFNγ_only) (Fig. 5B-C). We performed a series of analyses to identify the various pathways enriched in these gene sets from both lines to determine how ERK activation could lead to cell death. Pathway enrichment analysis clearly separated gene sets co-regulated by IFNγ and ERK from those regulated only by IFNγ (Fig. 5D). Pathways related to interleukin and interferon signaling, antigen processing and presentation, and antiviral response were all enriched in the IFNγ_only regulated set in both M230 and M238 lines. In contrast, three pathways related to integrated stress response activation were enriched in gene sets co-regulated by IFNγ and ERK (M238_IFNγ_ERK). These stress-related pathways were only enriched in M238 cells but not M230 cells, providing an indication of differences that may lead to a dependence on ERK for cell death induction. Similar results were found using the HALLMARK gene sets (Fig. S11A), where the IFNγ_ERK gene set was enriched for genes belonging to the unfolded protein response pathway only in the M238 line, again highlighting that stress response genes were an essential component of the IFNγ_ERK co-regulated gene set in these cells. Transcription factor binding site analysis showed similar results (Fig. 5E). IFNγ_only gene promoters were enriched for IRF1 and ISRE binding sites. This was true for M230 and M238 cell lines, indicating that the regulation of these genes was common to both. However, IFNγ_ERK genes were enriched for SRF, CHOP, and CEBPB sites in their promoters. Enrichment of SRF sites in this set of genes is indicative of regulation by ERK signaling, which activates the SRF-mediated transcription of target genes [22]. On the other hand, both *CHOP* and *CEBPB* sites indicate the activation of an integrated stress response program [23]. These sites were only enriched in the M238 line but not in the M230 line. An analysis of 24 stress-response-related genes, including DR5 (*TNFRSF10B*) and NOXA (*PMAIP1*), that are responsible for cell death induction following unresolved cellular stress [23–27], showed that stress-related genes were induced in M238 cells following IFNγ exposure (Fig. 5F, right panel). This induction was completely inhibited by culture with ulixertinib, indicating that they were directly downstream of ERK activation. However, none of these genes were induced in the M230 line (Fig. 5F, left panel, and Fig. S12).

In all the comparisons, IFNγ_only gene set from M238 cells always clustered together with all three sets from the M230 cells. This indicates that ERK-regulated gene expression differentiates the IFNγ response in the M238 cell line. Additionally, the induction of stress response genes, including upregulation of DR5 and NOXA, characterizes the ERK-induced response in melanoma cells, in which cell death can be rescued by ulixertinib.

### IFNγ regulates cell cycle genes independent of ERK activity

Since ulixertinib only rescued cell death but not cell growth (Fig. 4A-C), we determined if cell cycle genes were regulated differently by IFNγ and ERK in the M238 line. K-means clustering of 746 genes co-regulated by IFNγ and ERK (Fig. 5A, M238_IFNγ_ERK) revealed four distinct regulatory patterns (Fig. S13A). Pathway enrichment analysis showed that cluster 6 was enriched for cell cycle genes, including *PCNA, CCNE2, TERT*, and *MCM10*, which were downregulated upon IFNγ exposure (Fig. S13B). Cyclin A2 (*CCNA2*), which narrowly missed the cutoff for differentially expressed genes, also showed the same pattern (Fig. S13C). Inhibition of ERK signaling also downregulated these genes, indicating that ERK positively regulates these genes while IFNγ represses their expression (Fig. S13A, see values for cluster 6). Since these genes were downregulated with DMSO plus IFNγ treatment (Fig. S13C, second sample) when ERK signaling was fully active, this pathway likely represents an ERK-independent mode of growth inhibition through cell cycle arrest. The gene promoters from this cluster were also enriched for E2F binding sites, indicating their involvement in cell cycle progression [28] (Fig. S13D). Taken together, our results indicate the presence of separate IFNγ-mediated cell death and cell cycle arrest pathways in melanoma cells.

### ERK activation induces cell death through stress induction

**Fig. 6:**
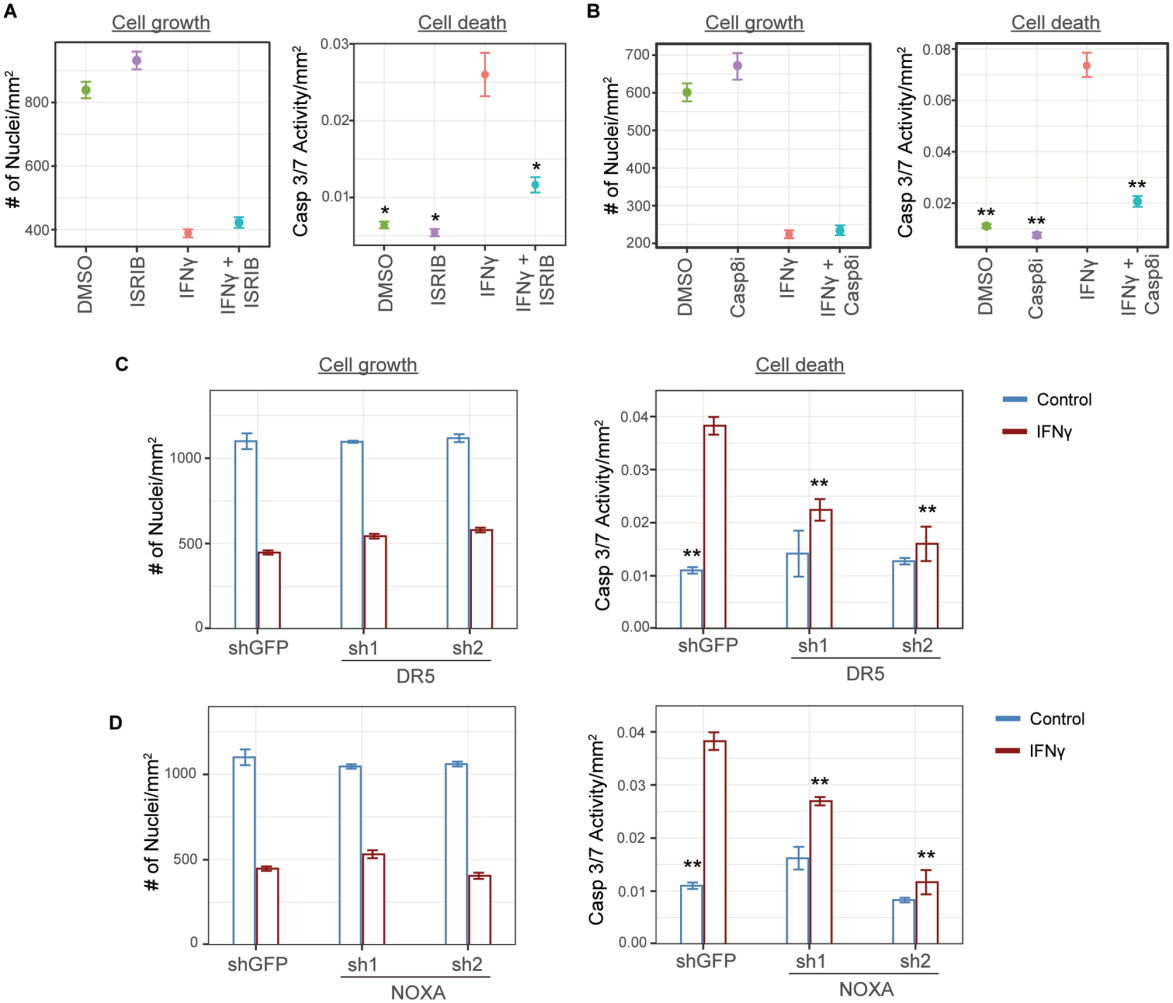
ERK induces cell death through the induction of stress response. M238 cells were treated in triplicates with **A**. ISRIB (1 µM), an inhibitor of stress response induction, or **B**. Z-IETD-FMK (50 µM), a Caspase 8 inhibitor (Casp8i), in the presence or absence of IFNγ. Cells were stained on day 5 and imaged using the IncuCyte Zoom instrument to enumerate total DNA-containing objects (left panels) and those with caspase 3/7 activity. Caspase activity counts were normalized to the total DNA-containing object counts, and means were plotted in the right panels. Error bars indicate SEM. Results are representative of 3 and 2 independent experiments, respectively. Stable cell lines expressing shRNAs against GFP (control) and **C**. DR5, or **D**. NOXA, were treated with or without IFNγ in triplicate wells per condition. Cells were stained as in panel A on day 5 of treatment, and mean values for cell growth (left) and cell death (right) were plotted for each condition. Error bars indicate SEM. Results are representative of 3 independent experiments. Statistically significant differences in cell death as compared to the IFNγ-treated sample (panels A and B) and shGFP plus IFNγ sample (panels C and D) are indicated by an asterisk: * *P* < 0.05, ** *P* < 0.01. See also Fig. S14

Pathways related to the induction of the integrated stress response were among those that differentiated ulixertinib-rescued M238 cells (Fig. 5D and Fig. S11A) from the M230 line. Hence, we tested the importance of stress induction in apoptosis. We cultured cells with an inhibitor of the integrated stress response, ISRIB [29], in the presence or absence of IFNγ (Fig. 6A). ISRIB alone did not affect cell growth (Fig. 6A, left panel), but it significantly blocked IFNγ-induced apoptosis (Fig. 6A, right panel). Again, similar to the results from ulixertinib experiments, ISRIB did not rescue cell growth. Both DR5 and NOXA induce cell death following unmitigated cellular stress [23, 24, 26, 27, 30, 31]. In the case of ER stress, DR5 undergoes ligand-independent activation through receptor aggregation leading to Caspase-8 activation resulting in subsequent Caspase 3/7 activation and apoptosis [27]. Thus, we first tested if a specific inhibitor of Caspase-8 (Casp8i) can block IFNγ-induced cell death. Caspase-8 inhibitor Z-IETD-FMK inhibited cell death (Fig. 6B), similar to ulixertinib (Fig. 4A) and ISRIB (Fig. 6A). Finally, we generated M238 lines stably expressing DR5 and NOXA shRNAs to knock down the expression of these genes (Fig. S14). While IFNγ induced apoptosis, as seen by an increase in Caspase 3/7 activity in shGFP-expressing control cells, the number of apoptotic cells was significantly lower in the lines expressing DR5 and NOXA shRNAs (Fig. 6C and D, respectively). Thus, our experiments confirmed the role of DR5 and NOXA in apoptosis, as predicted by the RNAseq analysis.

Collectively, these data situate ERK activation, stress response induction, and DR5 and NOXA activation in the same pathway that leads to cell death and provides experimental evidence for the connection between IFNγ-ERK signaling, subsequent stress response induction, and apoptosis.

## Discussion

The strong antitumor activity of IFNγ is crucial for the success of immune checkpoint blockade (ICB) therapy [1, 18, 32–34]. Disruption of IFNγ signaling makes tumors resistant to anti-PD-1/L1 treatment proposed to be mediated by evasion of the antiproliferative effects of this cytokine [35]. Resistance to IFNγ may be widespread, as shown by our results with melanoma lines. A recent study also found more than half of the tested cancer cell lines to be resistant to IFNγ-mediated growth inhibition [36]. In a preclinical model, IFNGR2 and JAK1 KO cells outgrew wild-type cells in a mixed tumor model [37]. These studies provide a rationale and highlight the clinical significance of our work in delineating the growth inhibition pathway. Our results demonstrate that ERK activation and downstream induction of stress response are essential events that lead to melanoma cell death. A recent study [36] also found both DR5 and NOXA among the top hits in a screen spanning multiple cancer cell lines, making it likely that the ERK-mediated pathway we describe here is also functional in other tumor types.

Although only *ERK2* (not *ERK1*) was a hit in our CRISPR screens, we hypothesize that an overall increase in ERK1/2 activity is important for the induction of cell death. This is based on the following considerations: (i) M238, the *BRAF* V600E mutant melanoma cell line used in our CRISPR screens, has a much higher expression of *ERK2* than *ERK1*, which is also reflected in the ratio of activated levels of these proteins. Thus, only ERK1 deletion may not cause a sufficient reduction in global ERK activity to disrupt the cell death pathway. (ii) Human *ERK1* and *ERK2* proteins have > 85% sequence homology. (iii) *ERK1* can fully substitute *ERK2* during mouse embryonic development [38]. (iv) Lastly, both *ERK1* and *ERK2* overexpression results in cell death of human melanoma lines [39]. Despite this, some studies have demonstrated a specific requirement for ERK1 or ERK2 [40, 41]. Additional experiments will be needed to determine if ERK2 is specifically needed for cell death induction in melanoma cells.

Despite clear evidence for the involvement of ERK, the mechanism of crosstalk between ERK and IFNγ signaling and how it leads to the induction of stress response in melanoma cells remains to be elucidated. Constitutive ERK signaling was shown to downregulate IFNAR1 expression in BRAF mutant melanomas, which made the tumors resistant to type I interferon-mediated growth inhibition [42]. On the other hand, our gene expression analysis revealed a positive role for ERK in the regulation of chemokine and IFNGR2 expression downstream of IFNγ signaling in M238 cells. Thus, the interaction between IFN and ERK signaling pathways is complex, and more work is needed to understand how it shapes antitumor immunity. Also, while we demonstrated that the IFNγ-ERK cell death pathway is functional in a majority of the tested human melanoma lines, we did not investigate each line in detail. Hence it is possible that other parallel pathways are involved in cell death induction in some of these lines.

ERK signaling is tightly regulated at multiple nodes with feedback regulatory mechanisms [43]. We propose a model in which IFNγ signaling leads to the hyperactivation of ERK in melanoma cells, leading to cell death (Fig. S15). Results from several groups corroborate our findings and proposed model based on ERK hyperactivation. DUSP4, a negative regulator of ERK activity, was found to be expressed at elevated levels in MAPK mutant melanomas [44]. Deletion of negative ERK regulators, including DUSP4, DUSP6, and PEA15, induced cell death in *BRAF* and *NRAS* mutant melanomas due to unrestrained activation of ERK [44–46]. Another study showed that overexpression of ERK1 or ERK2 leads to cell death of human melanoma cell lines [39]. Finally, two groups have demonstrated that hyperactivation of ERK signaling is responsible for cell death upon drug withdrawal in BRAF and MEK inhibitor-addicted melanoma cells [47, 48].

## Conclusions

Our study demonstrates a novel cell death pathway mediated by the IFNγ-ERK signaling axis in melanoma cells. IFNγ can escape the immune synapse and induce signaling in tumor cells several layers away from the site of secretion [49, 50]. Given its ability to penetrate deep inside the tumor, modulating the IFNγ-ERK cell death pathway described here could be an effective strategy for controlling the tumor burden. Our findings also provide an opportunity to understand and overcome the resistance mechanisms in tumors that are impervious to IFNγ-mediated growth inhibition.

### Electronic supplementary material

Below is the link to the electronic supplementary material.

Supplementary Material 1

Supplementary Material 2

Supplementary Material 3

Supplementary Material 4

## Data Availability

GFP, DR5, and NOXA shRNA constructs generated in this study are available upon request. Sequencing data from the CRISPR/Cas9 screen and RNA-seq experiments are available through the Gene Expression Omnibus (GEO) database using the accession number GSE235238 (https://www.ncbi.nlm.nih.gov/geo/query/acc.cgi?acc=GSE235238).

## Abbreviations

AUC: Area under the curve
CRISPR: Clustered regularly interspaced short palindromic repeats
DMSO: Dimethyl sulfoxide
DR5: Death receptor 5
ERK: Extracellular signal-regulated kinase
FBS: Fetal bovine serum
ICB: Immune checkpoint blockade
IFNγ: Interferon-gamma
KD: Knockout
KO: Knockdown
MAPK: Mitogen-activated protein kinase
MHC: Major histocompatibility complex
PMA: phorbol 12-myristate 13-acetate
SEM: Standard error of the mean
TCGB: Technology Center for Genomics and Bioinformatics
Ulix: Ulixertinib
