# Correction: ERK mediates interferon gamma-induced melanoma cell death

**DOI:** 10.1186/s12943-024-01949-5

**Published:** 2024-02-05

**Authors:** Ameya Champhekar, Rachel Heymans, Justin Saco, Guillem Turon Font, Cynthia Gonzalez, Anne Gao, John Pham, June Lee, Ryan Maryoung, Egmidio Medina, Katie M. Campbell, Daniel Karin, David Austin, Robert Damioseaux, Antoni Ribas

**Affiliations:** 1 Division of Hematology-Oncology, Department of Medicine, University of California, Los Angeles, Los Angeles, CA 90095 USA; 2 Department of Molecular and Medical Pharmacology, University of California, Los Angeles, CA 90095 USA; 3 California NanoSystems Institute, University of California, Los Angeles, CA 90095 USA; 4 Jonsson Comprehensive Cancer Center, Los Angeles, CA 90095 USA; 5 Department of Bioengineering, Samueli School of Engineering, University of California, Los Angeles, CA 90095 USA; 6 Division of Surgical Oncology, Department of Surgery, University of California, Los Angeles, Los Angeles, CA 90095 USA; 7 Parker Institute for Cancer Immunotherapy, San Francisco, CA 94129 USA

**Correction: Mol Cancer 22, 165 (2023)**

10.1186/s12943-023-01868-x

Following publication of the original article [1], the authors reported that an author’s last name that appeared in the published online version is incorrect. Robert Damioseaux should be Robert Damoiseaux.
