# Supplementary material for: ERK mediates interferon gamma-induced melanoma cell death
Source: Mol Cancer. 2023 Oct 6;22:165. doi: 10.1186/s12943-023-01868-x (PMC10557262; doi:10.1186/s12943-023-01868-x)
Supplement: Supplementary file 1 — Supplementary Material 1 [file 12943_2023_1868_MOESM1_ESM.docx]

**SUPPLEMENTARY FIGURES AND FIGURE LEGENDS**


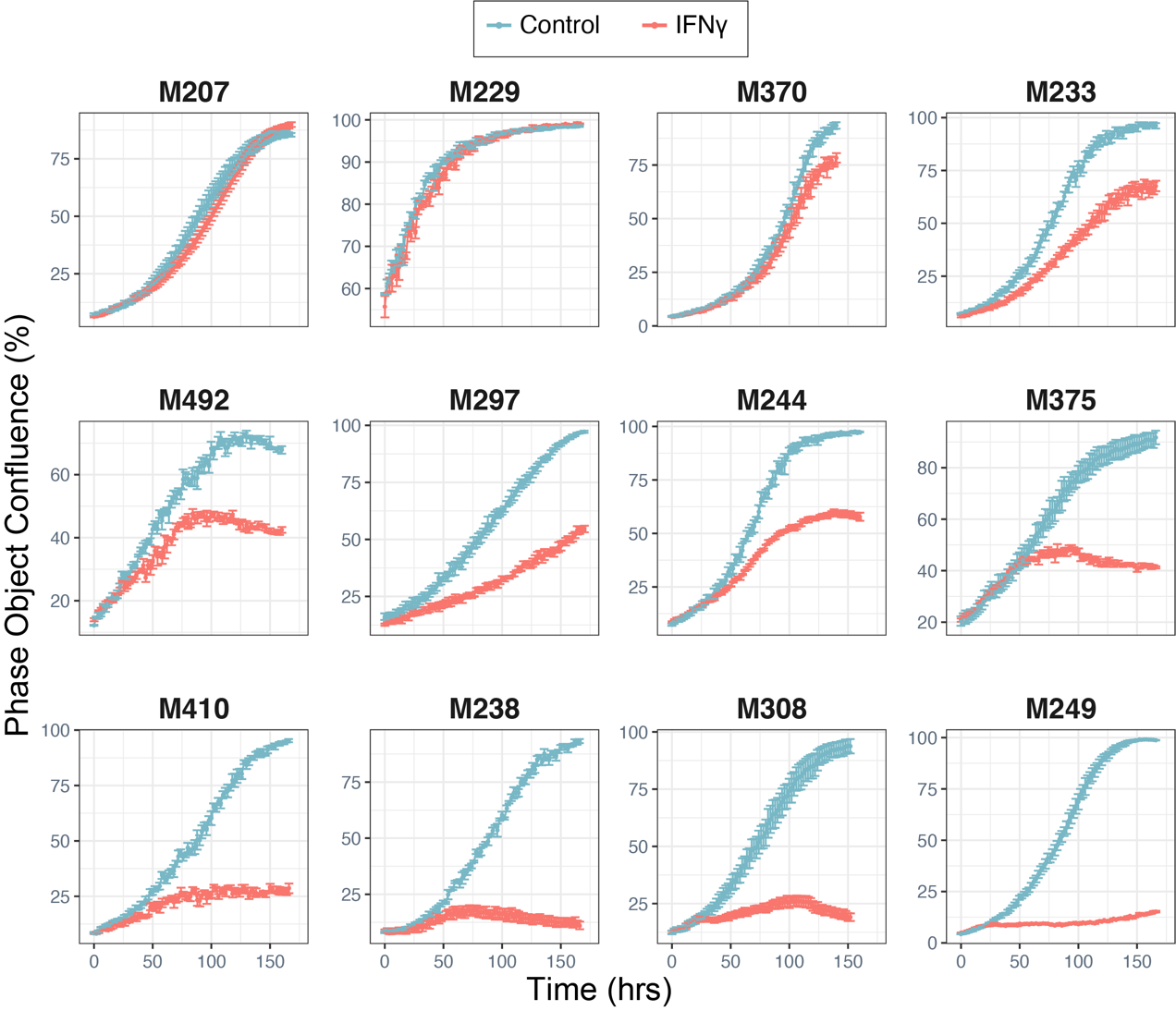


**Fig. S1.** Melanoma lines were treated with or without IFNγ in triplicate, and cell growth was monitored for 7 days in an IncuCyte live imaging experiment. The mean confluency of triplicate wells for each condition was plotted over the course of the experiment for IFNγ-resistant (top row), intermediate (middle row), and sensitive (bottom row) lines from the 31-line set presented in Fig 1A. Error bars indicate SEM.


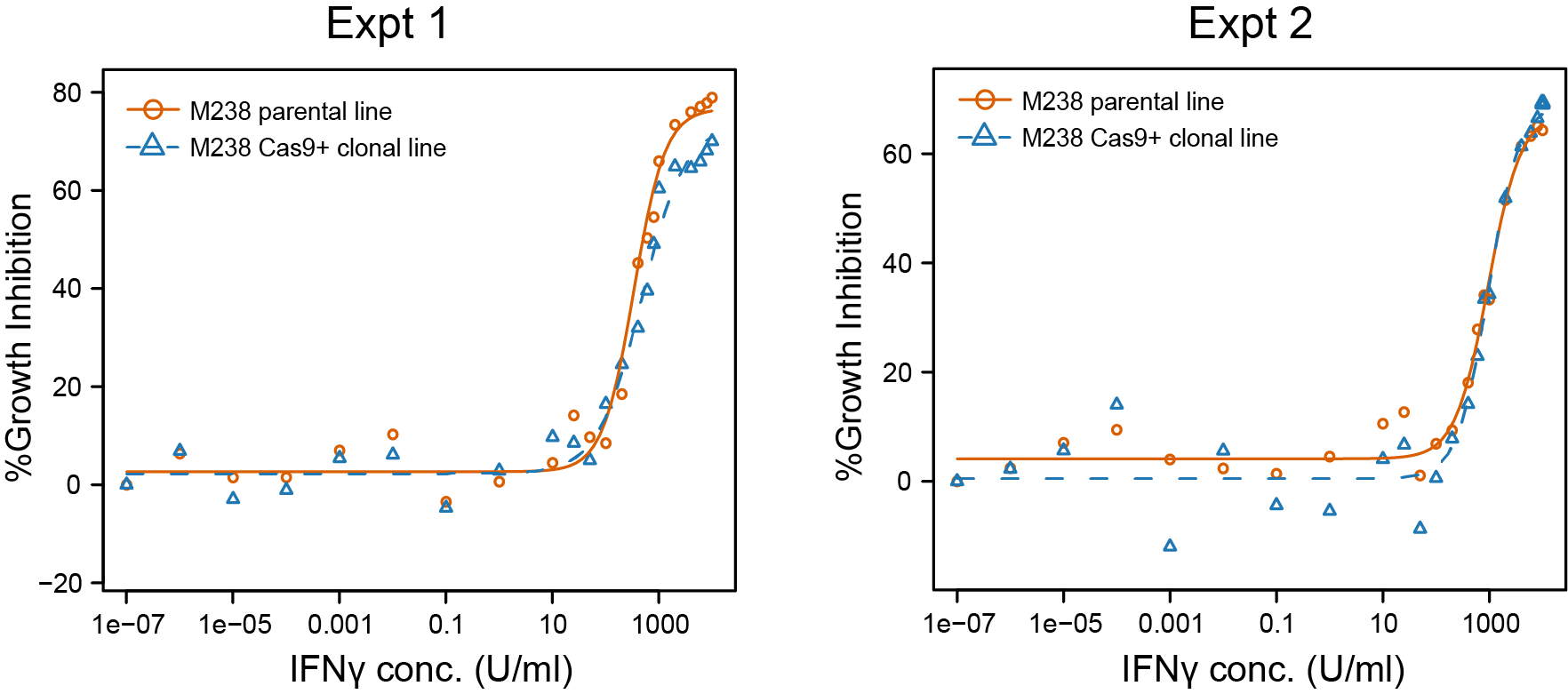


**Fig. S2. Cas9 expressing M238 clonal line used for CRISPR screening is representative of the M238 parental line for growth inhibition studies.** M238 parental line and Cas9 expressing clonal line were both treated with IFNγ doses ranging from 10^-7 to 10^4 Units/ml, and dose-response curves were plotted for two experiments, as shown in the figure.


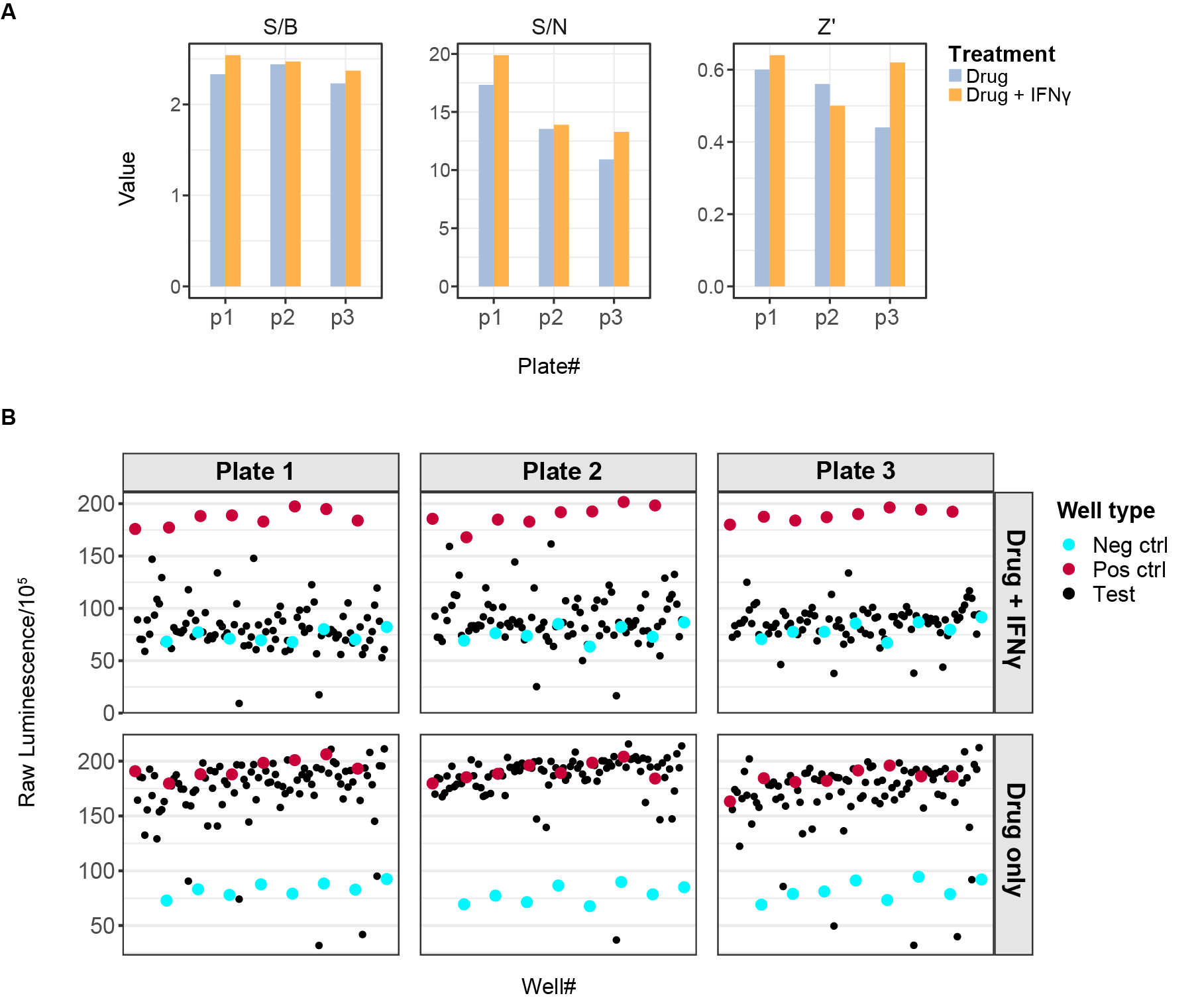


**Fig. S3. Assay metrics and raw data for the confirmatory screen. A.** Signal-to-Background ratio (S/B), Signal-to-Noise ratio (S/N), and Z’ score for all 6 plates (3 x Drug only and 3 x Drug plus IFNγ) that were a part of the confirmatory screen. **B.** Raw luminescence for each individual plate run for the confirmatory screen (lower panels). Luminescence values from negative control (IFNγ) and positive control (DMSO) wells are shown as blue and red dots, respectively. Values for all the test compounds are shown as black dots.

**
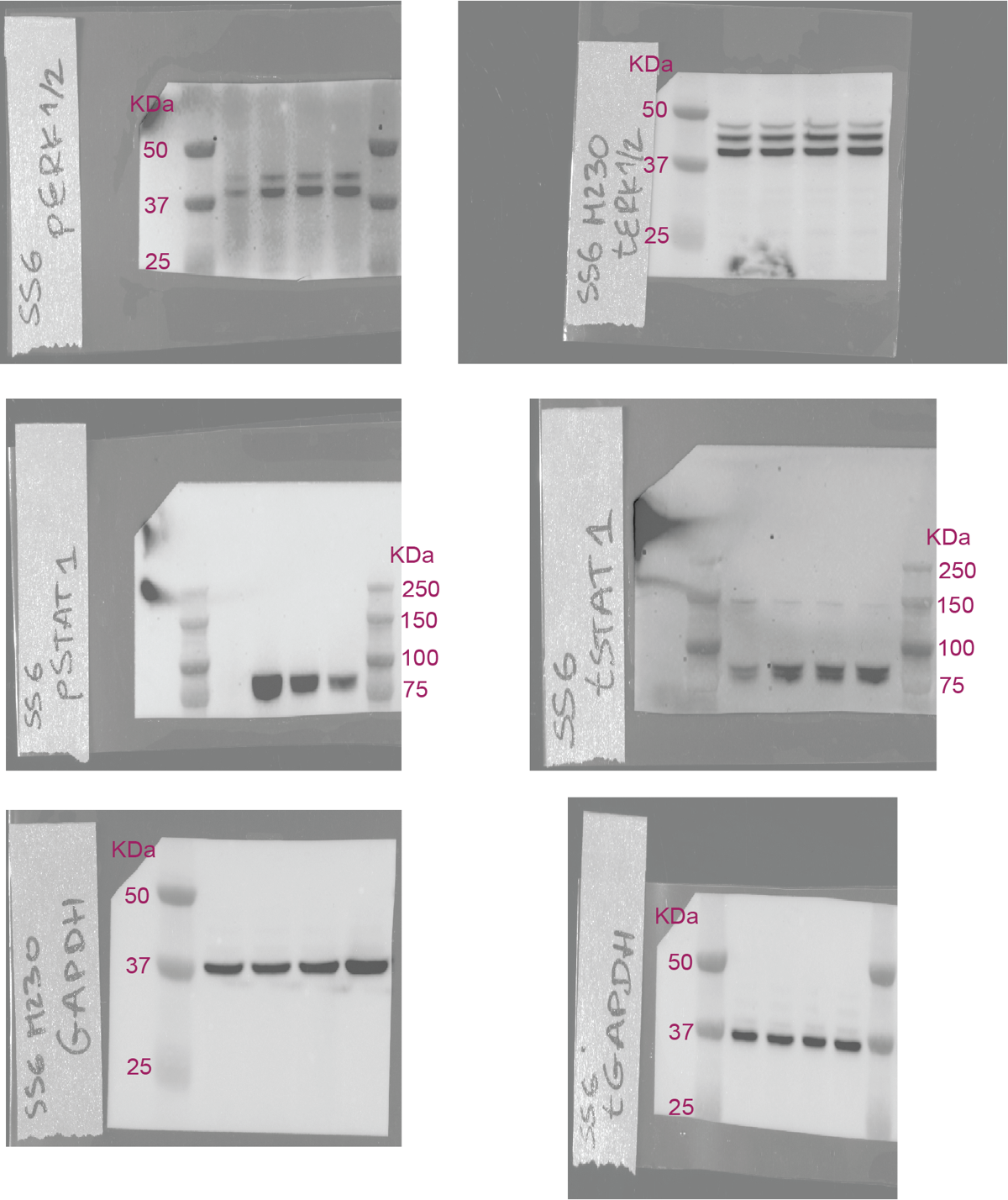
**

**Fig. S4. Full blots for each row in Fig. 3A.** Each blot was serially imaged in the same position in chemiluminescent and colorimetric modes using the Bio-Rad ChemiDoc MP Imaging System. The two images were merged using the onboard software provided by the manufacturer and presented here to show the position of bands with respect to the Precision Plus Protein Dual Color ladder (Bio-Rad, Cat# 1610374). Molecular weights of protein ladder are labeled in red font.

**
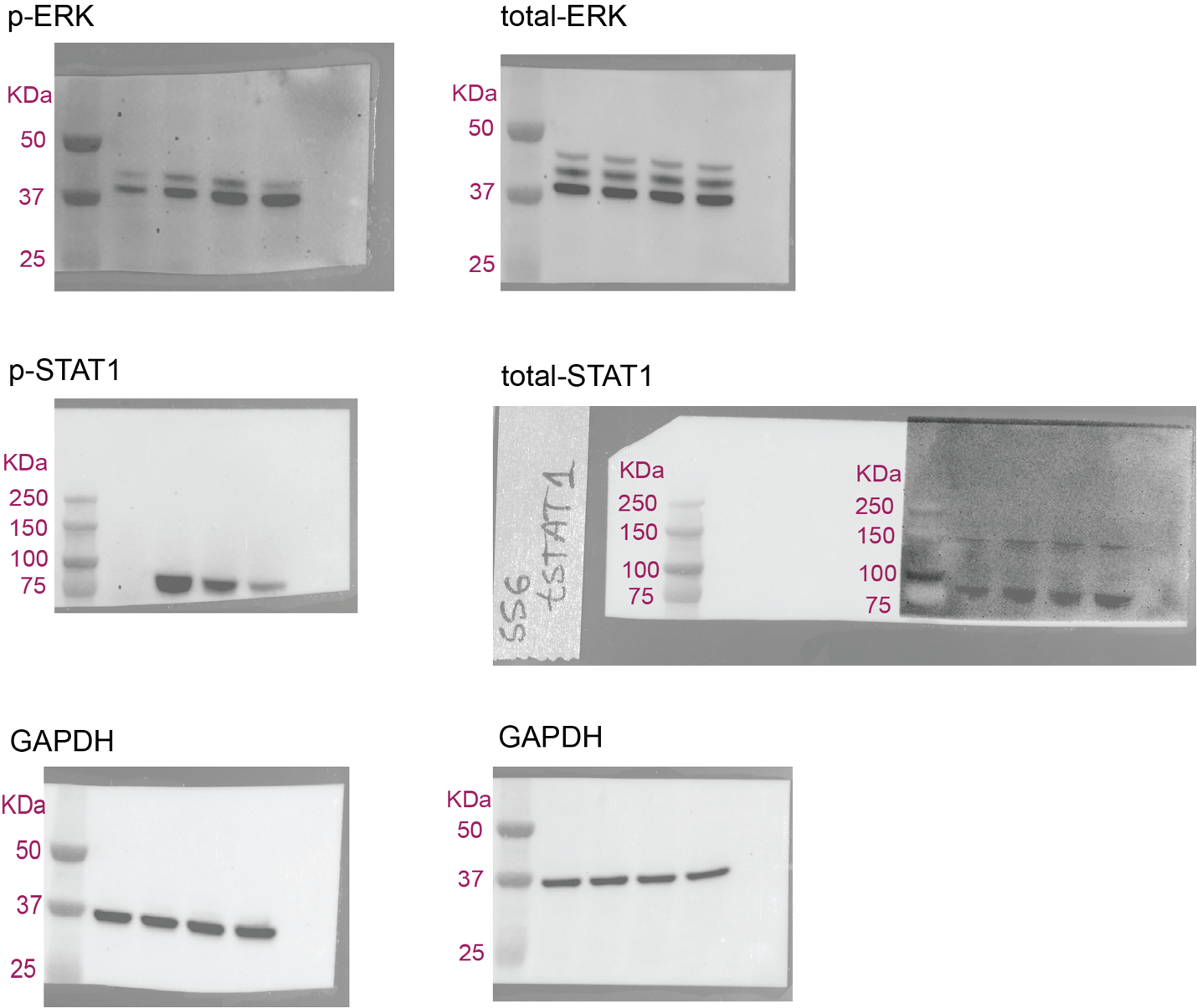
**

**Fig. S5. Full blots for each row in Fig. 3B.** Each blot was serially imaged in the same position in chemiluminescent and colorimetric modes using the Bio-Rad ChemiDoc MP Imaging System. The two images were merged using the onboard software provided by the manufacturer and presented here to show the position of bands with respect to the Precision Plus Protein Dual Color ladder (Bio-Rad, Cat# 1610374). Molecular weights of protein ladder are labeled in red font.

**
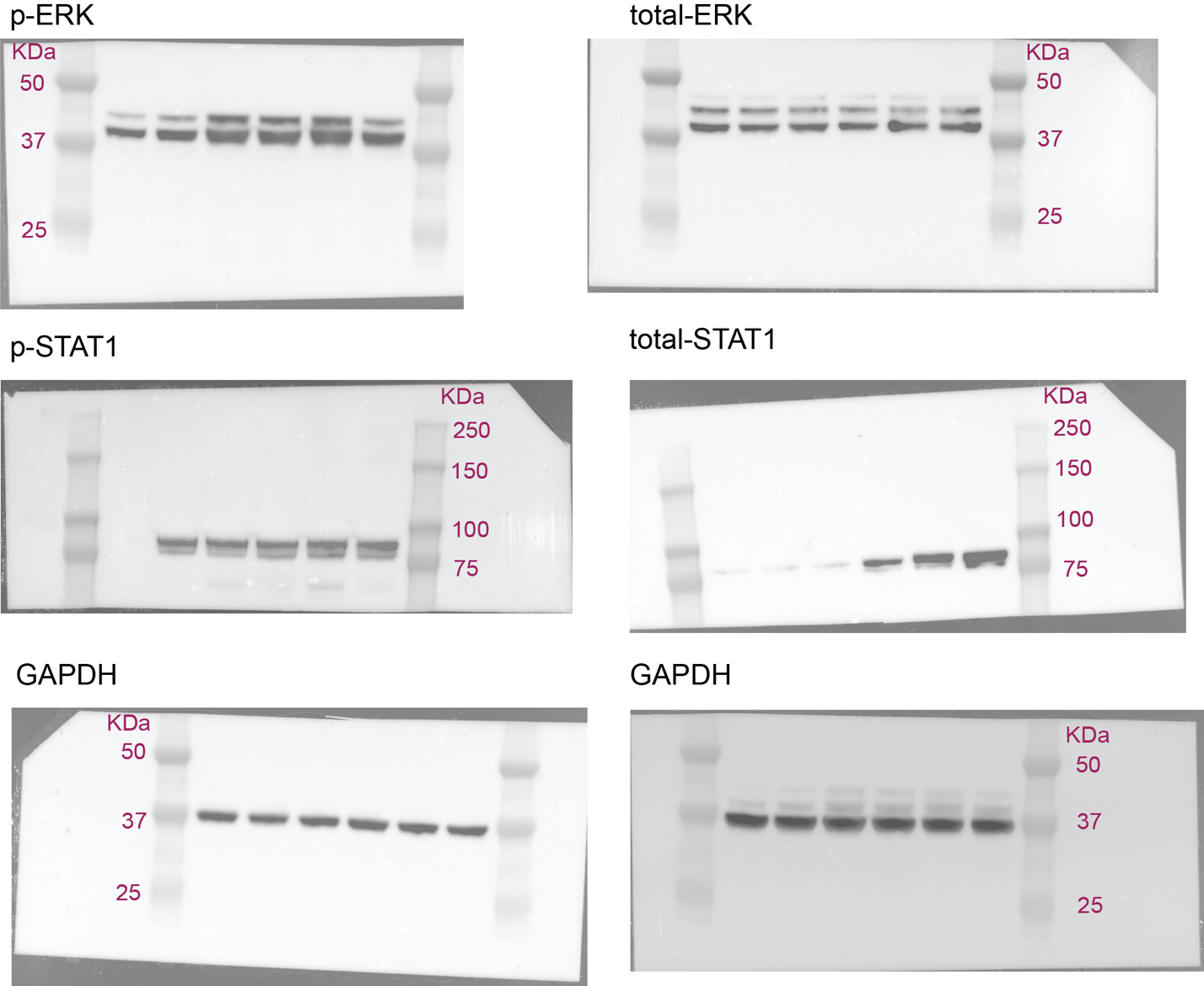
**

**Fig. S6. Full blots for each row in Fig. 3C.** Each blot was serially imaged in the same position in chemiluminescent and colorimetric modes using the Bio-Rad ChemiDoc MP Imaging System. The two images were merged using the onboard software provided by the manufacturer and presented here to show the position of bands with respect to the Precision Plus Protein Dual Color ladder (Bio-Rad, Cat# 1610374). Molecular weights of protein ladder are labeled in red font.

**
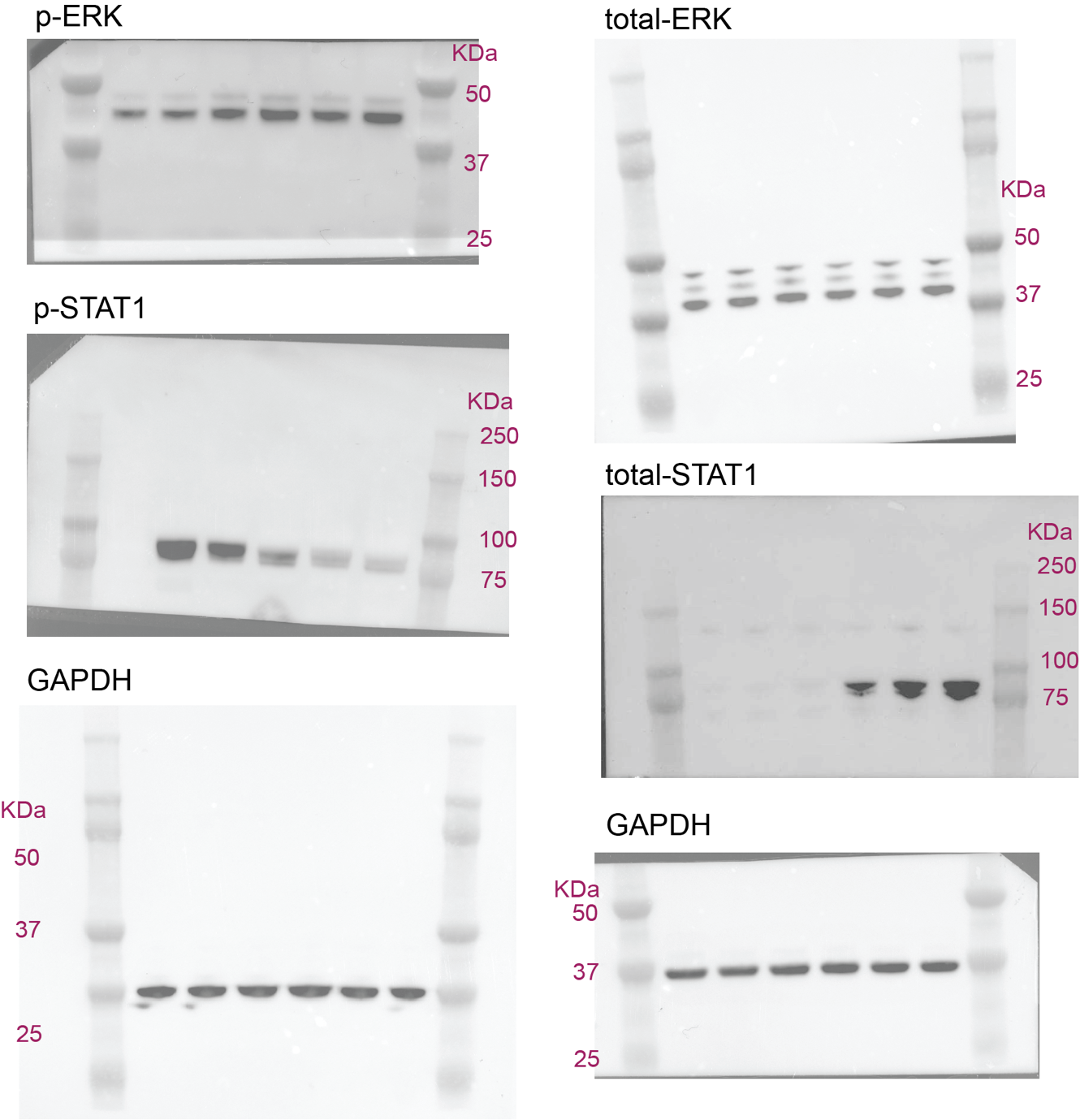
**

**Fig. S7. Full blots for each row in Fig. 3D.** Each blot was serially imaged in the same position in chemiluminescent and colorimetric modes using the Bio-Rad ChemiDoc MP Imaging System. The two images were merged using the onboard software provided by the manufacturer and presented here to show the position of bands with respect to the Precision Plus Protein Dual Color ladder (Bio-Rad, Cat# 1610374). Molecular weights of protein ladder are labeled in red font.

**
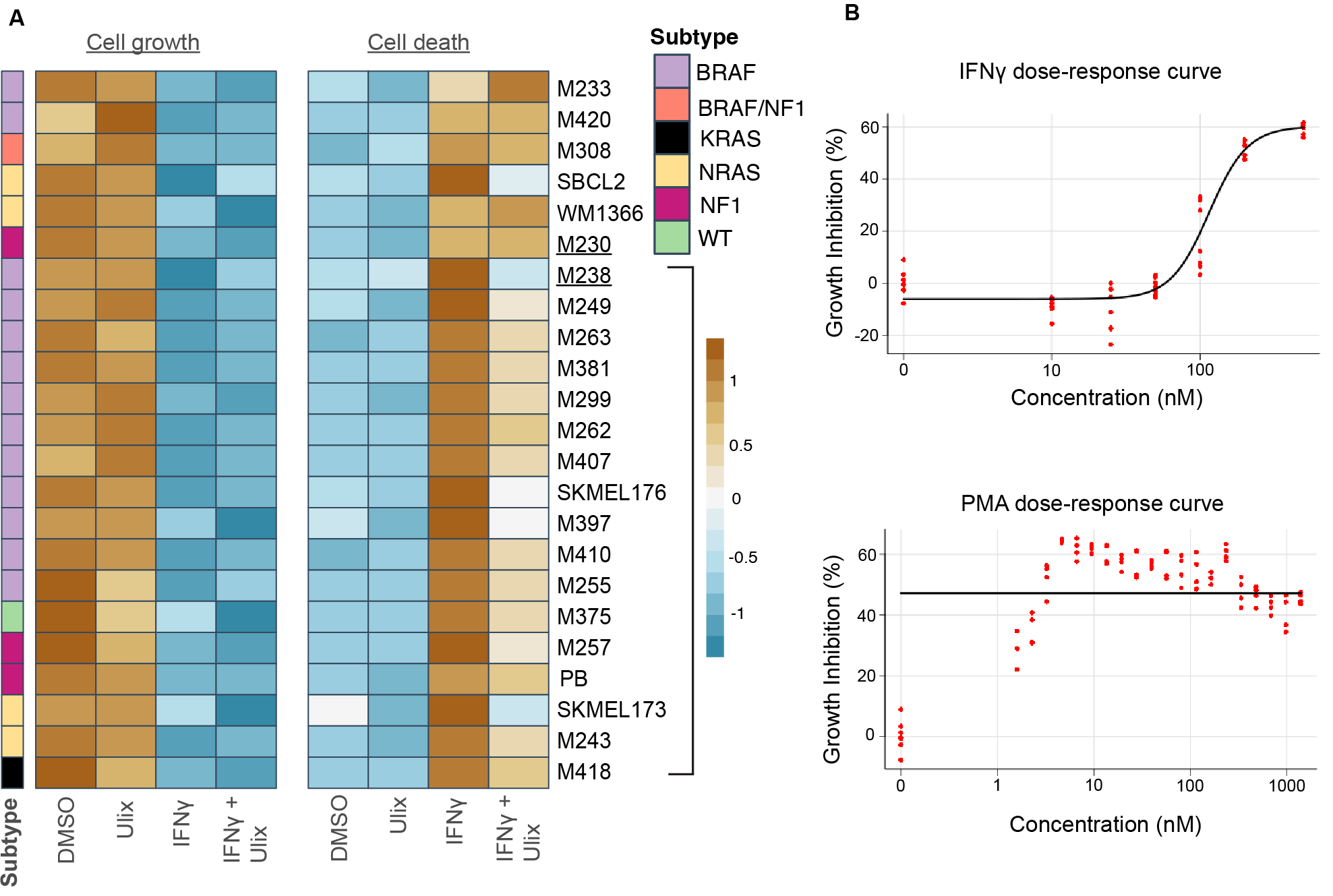
**

**Fig. S8. A.** Heatmaps for the second independent dataset generated using IncuCyte live imaging data as in Fig. 4C. Heatmap labels for M230 and M238 lines, compared in an RNA-seq experiment in Fig. 5, are indicated with an underline. **B.** Dose-response curves for growth inhibition of M238 cells with IFNγ (top panel) and PMA (lower panel).

**
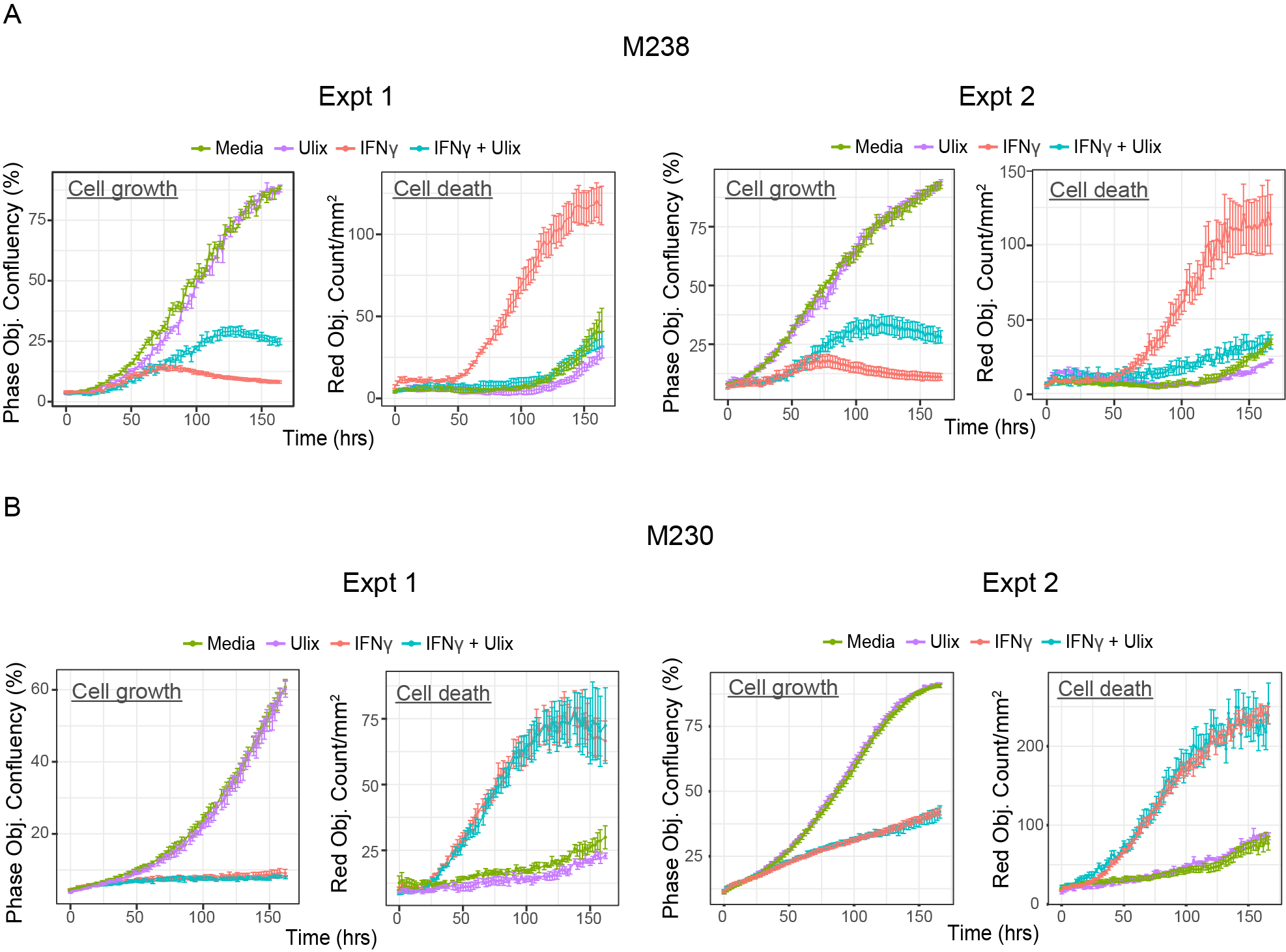
**

**Fig. S9. Rescue of IFNγ-mediated cell death with ulixertinib treatment for M230 and M238 lines.** Results from 2 independent experiments are presented in the top and bottom rows for **A.** M238 and **B.** M230 melanoma lines, respectively. Cells were plated with the indicated treatments in triplicate wells per condition. The Cytotox Red dye, which stains dead cells, was added to all wells. For each experiment, plots show changes in confluence (left panel) and dead cell count (right panel) over time obtained through an IncuCyte live imaging experiment. Error bars indicate the SEM for each set of triplicate wells at each time point. These data show that the ERK inhibitor ulixertinib blocks cell death induction in IFNγ-treated M238 lines but not for the M230 line.

**
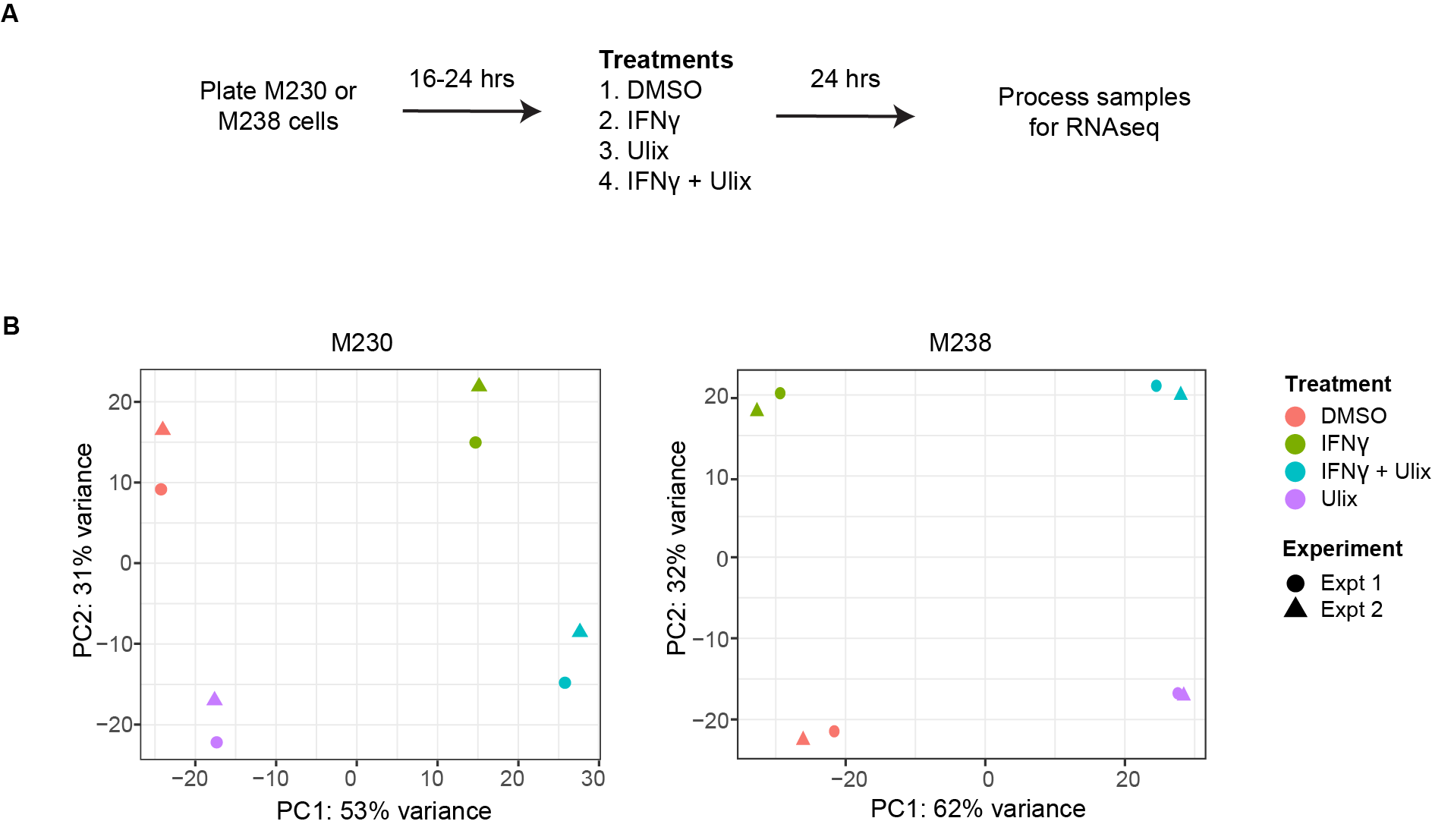
**

**Fig. S10. RNAseq experimental setup. A.** Workflow for treatment of cells and RNAseq sample generation. **B.** RNAseq count data for each line from two independent experiments was used to perform Principal Component Analysis using the R package DeSeq2, and the resulting data were plotted for each line.

**
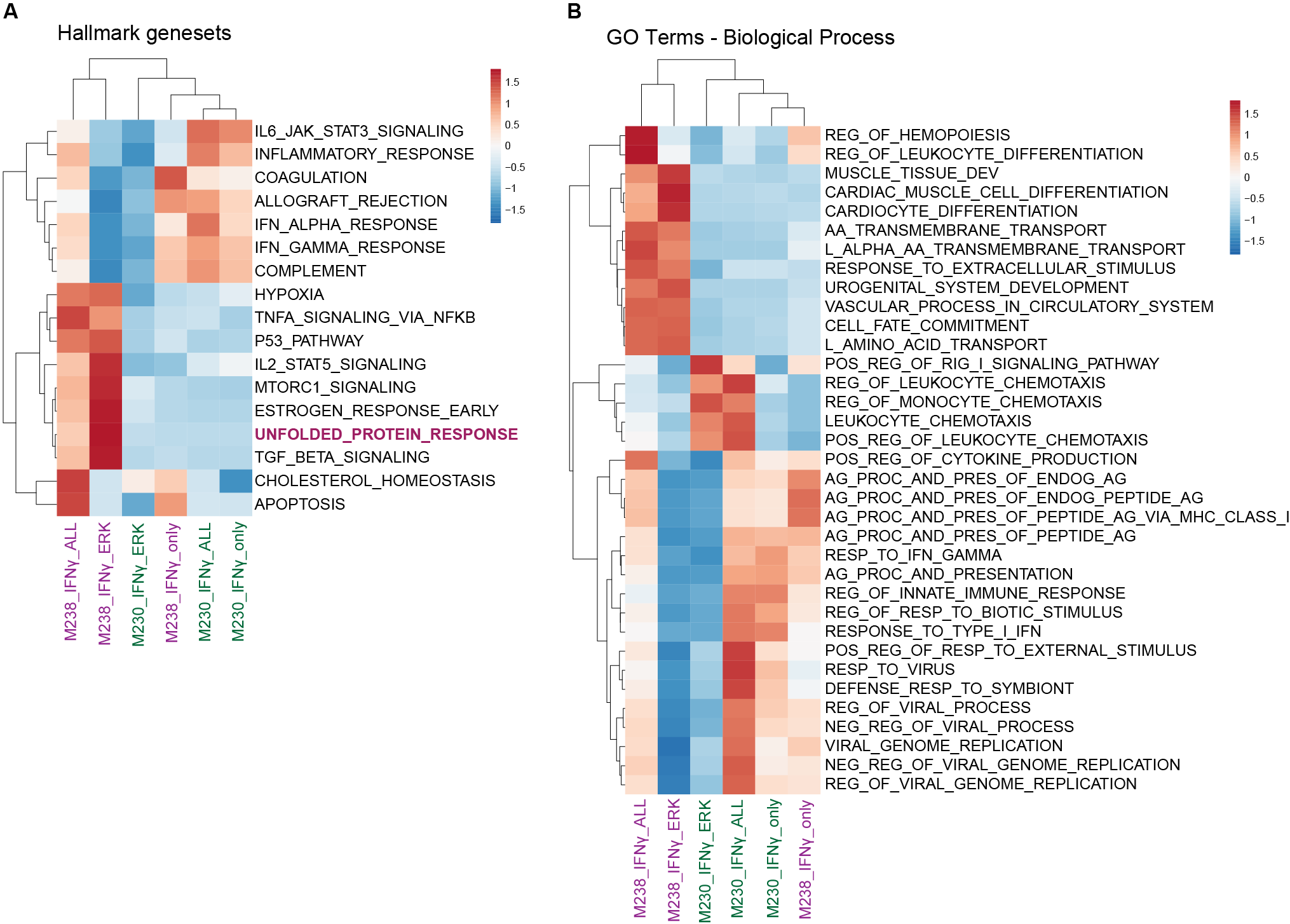
**

**Fig. S11. Enrichment analysis for various gene subsets.** Gene sets from the table in Fig. 5C were used to determine the enrichment of hallmark gene sets and gene ontology biological process (GO BP) terms in each subset. Heatmaps were generated using the negative log10 of adjusted p-values for a union set of the top 10 enriched **A.** hallmark gene sets and **B.** GO BP terms for each gene set. Gene sets for M230 line are labeled in green, while those for M238 are labeled in purple color. The hallmark gene set related to stress response induction is shown in red.


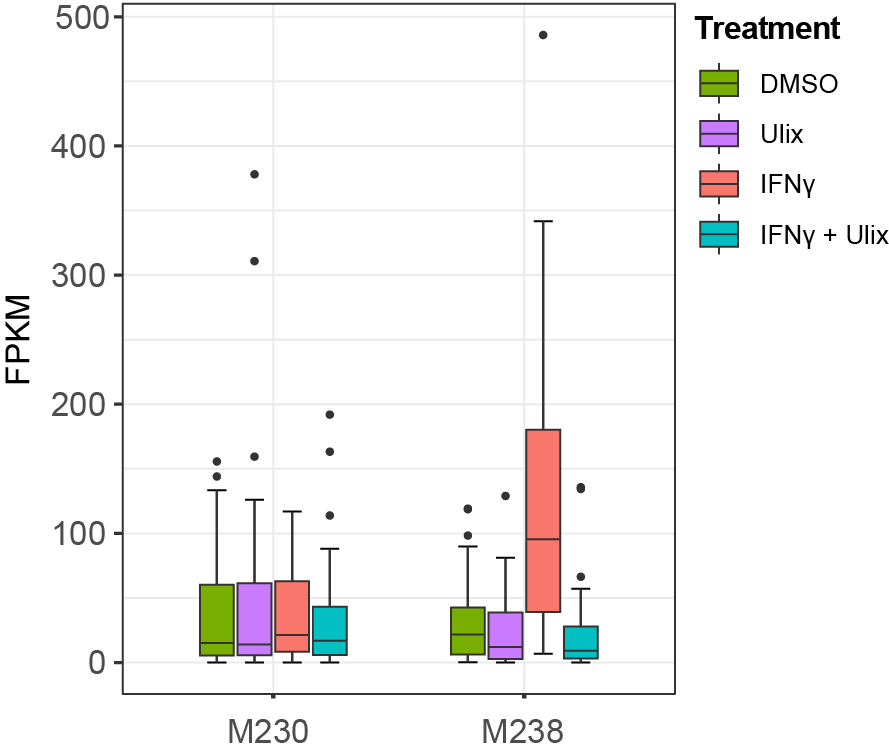


**Fig. S12. Stress response genes are not upregulated in M230 cells.** FPKM (**F**ragments **P**er **K**ilobase of transcript per **M**illion mapped reads) values for the 24 stress-response-related genes in Fig. 5F were plotted for each treatment and line. While the basal level of expression is uniformly low in both lines, only M238 line shows upregulation of these genes in response to IFNγ treatment.

**
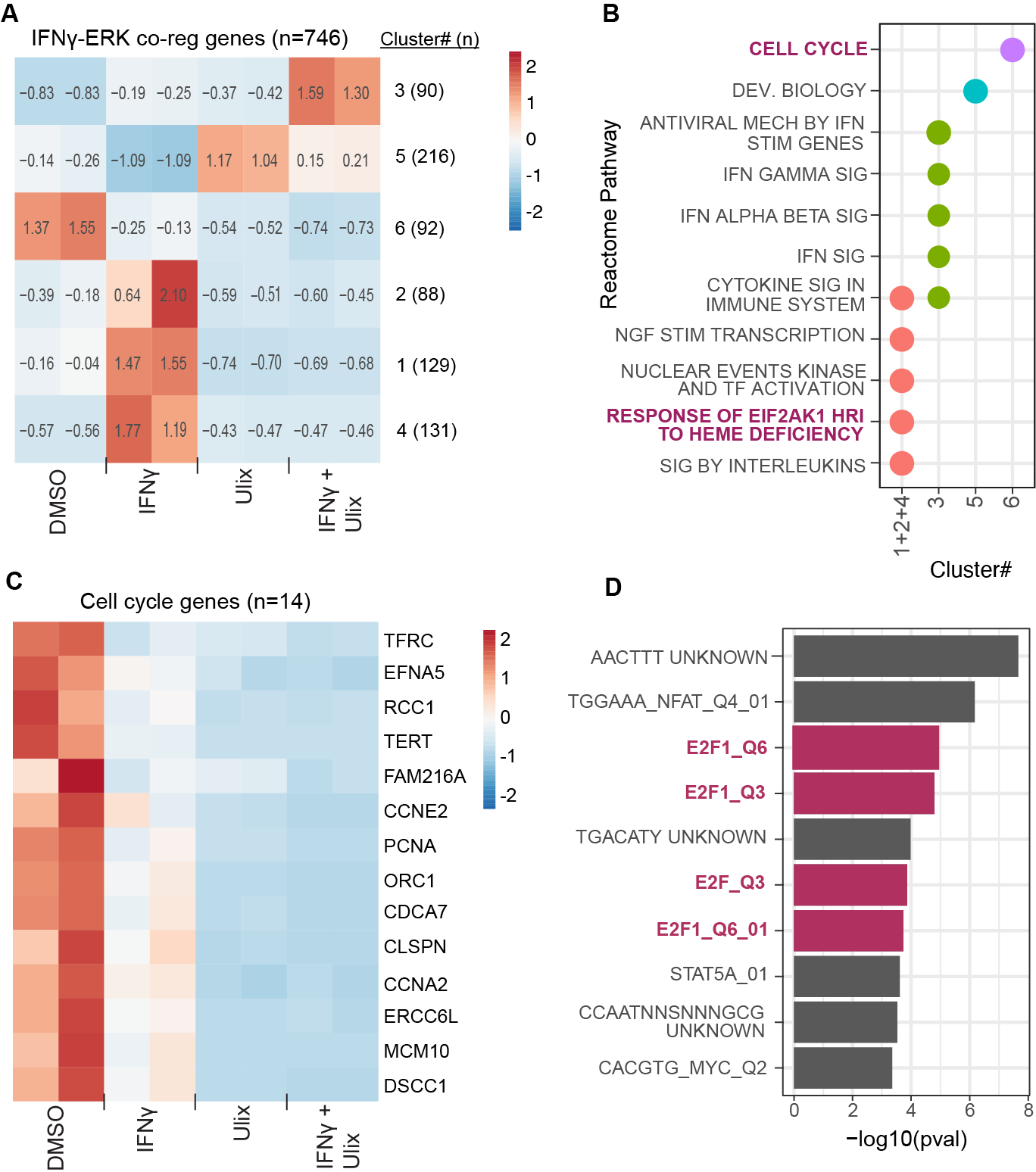
**

**Fig. S13. IFNγ represses cell cycle genes in the M238 line through an ERK-independent pathway. A.** K-means clustering analysis of 746 IFNγ-ERK co-regulated genes in the M238 line (M238_IFNγ_ERK). Numbers on the heatmap indicate the fold change values relative to the mean of each row. Cluster number and the number of genes in each cluster (n, numbers in brackets) are shown to the right. The two columns per sample represent data from two independent experiments. **B.** Genes from the clusters indicated on the x-axis were used for pathway enrichment analysis. Genes from clusters 1, 2, and 4 were pooled together for this analysis as they are regulated in a similar manner. The y-axis shows the top 5 enriched pathways for each cluster. Terms related to the cell cycle and a pathway related to stress response are labeled in red text. **C.** Heatmap using FPKM expression values for all cell cycle genes in cluster 6. The two columns per sample represent data from two independent experiments. **D.** Transcription factor sites enriched in the gene promoters from cluster 6. Red bars indicate the enrichment of E2F sites in these promoters.

**
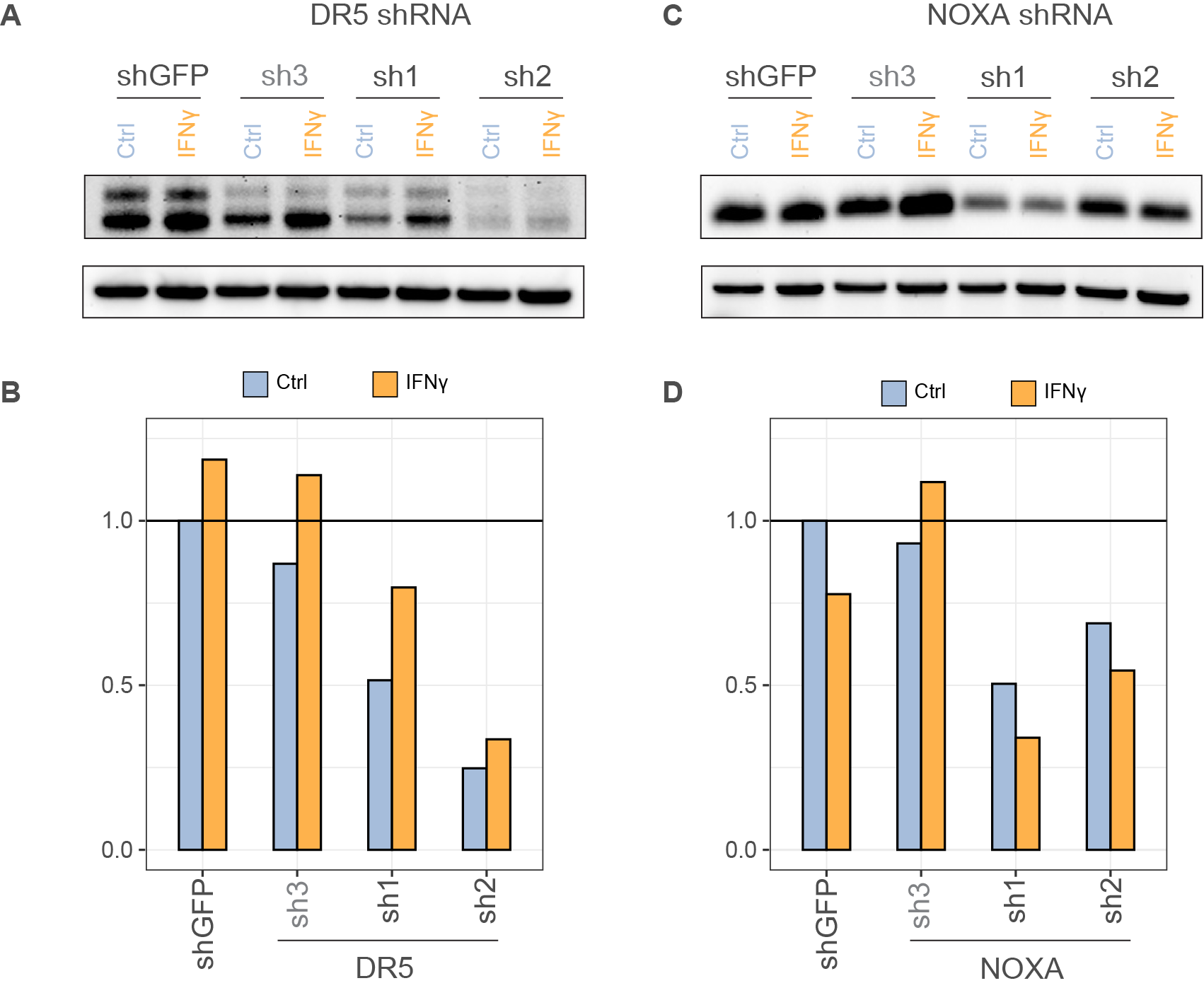
**

**Fig. S14. DR5 and NOXA knockdown in stable shRNA lines. A.** Stable lines expressing GFP or DR5 shRNA 1-3 were stimulated with IFNγ for 24 hours, and DR5 protein expression was determined by Western blotting. DR5 sh3 was not used in further experiments as it showed a low knockdown efficiency. **B.** Western blot bands from A were quantified and normalized to the shGFP control sample, and the resulting values were plotted. **C.** Stable lines expressing GFP or NOXA shRNA 1-3 were stimulated with IFNγ for 24 hours, and NOXA protein expression was determined by Western blotting. NOXA sh3 was not used in further experiments as it did not show any knockdown. **D.** Western blot bands from C were quantified and normalized to the shGFP control sample, and the resulting values were plotted.

**
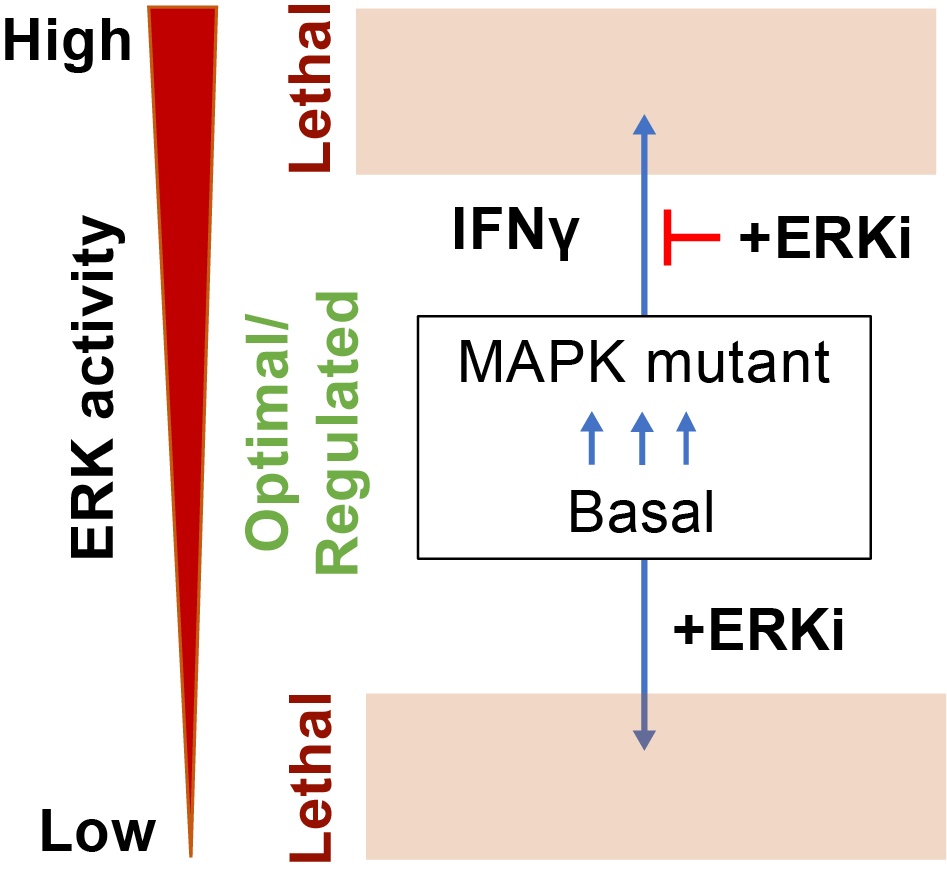
**

**Fig. S15. Proposed model for the regulation of cell fate by ERK activity levels.** Negative feedback regulation maintains ERK activity in an elevated but “optimal” range in MAPK mutant cancers. This level of signaling is conducive to transformation but not high enough to induce cell death. Based on our findings, we propose a model where IFNγ signaling pushes ERK activity over this threshold to induce cell death. At low concentrations, ERKi inhibits IFNγ-mediated ERK activation, which rescues cell death. However, at high ERKi concentrations, ERK activity is reduced to a very low level where cell survival is untenable irrespective of IFNγ treatment.
